# Effects of calcium channel blockers comparing to angiotensin-converting enzyme inhibitors and angiotensin receptor blockers in patients with hypertension and chronic kidney disease stage 3 to 5 and dialysis: A systematic review and meta-analysis

**DOI:** 10.1371/journal.pone.0188975

**Published:** 2017-12-14

**Authors:** Yen-Chung Lin, Jheng-Wei Lin, Mai-Szu Wu, Kuan-Chou Chen, Chiung-Chi Peng, Yi-No Kang

**Affiliations:** 1 Center for Evidence-based Medicine, Department of Education, Taipei Medical University Hospital, Taipei, Taiwan, R.O.C; 2 Graduate Institute of Clinical Medicine, College of Medicine, Taipei Medical University, Taipei, Taiwan, R.O.C; 3 Division of Nephrology, Department of Internal Medicine, Taipei Medical University Hospital, Taipei, Taiwan, R.O.C; 4 Department of Internal Medicine, School of Medicine, College of Medicine, Taipei Medical University, Taipei, Taiwan, R.O.C; The University of Tokyo, JAPAN

## Abstract

**Background:**

Calcium channel blocker (CCB) or two renin angiotensin aldosterone system blockades (RAAS), angiotensin-converting enzyme inhibitors (ACEIs) and angiotensin receptor blockers (ARBs), are major potent and prevalently used as initial antihypertensive agents for mild to moderate hypertension, but no uniform agreement as to which antihypertensive drugs should be given for initial therapy, especially among chronic kidney disease (CKD) patients.

**Design:**

A systematic review and meta-analysis comparing CCBs and the two RAAS blockades for hypertensive patients with CKD stage 3 to 5D. The inclusion criteria for this systematic review was RCT that compared the effects of CCBs and the two RAAS blockades in patients with hypertension and CKD. The exclusion criteria were (1) renal transplantation, (2) CKD stage 1 or 2, (3) combined therapy (data cannot be extracted separately). Outcomes were blood pressure change, mortality, heart failure, stroke or cerebrovascular events, and renal outcomes.

**Results:**

21 randomized controlled trials randomized 9,492 patients with hypertensive and CKD into CCBs and the two RAAS blockades treatments. The evidence showed no significant differences in blood presser change, mortality, heart failure, stroke or cerebrovascular events, and renal outcomes between CCBs group and the two RAAS blockades group. The publication bias of pooled mean blood presser change that was detected by Egger’s test was non-significant.

**Conclusions:**

CCBs has similar effects on long term blood pressure, mortality, heart failure, stroke or cerebrovascular events, and renal function to RAAS blockades in patients CKD stage 3 to 5D and hypertension.

## Introduction

Hypertension is a major contributor to mortality and cardiovascular disease in chronic kidney disease (CKD). Conflicting results have been reported regarding the benefits of blood pressure (BP) control, particularly in older individuals or those with CKD. In the Eighth Joint National Committee (JNC 8) guidelines[1] and a large randomized controlled trial (RCT) that showed a clinically considerable reduction in cardiovascular events and mortality in the intensive BP-lowering group[2]. However, the effects of BP control were non-significant in terms of renal outcomes, such as dialysis and renal function. In addition, the percentage of individuals with deteriorating renal function during this trial was almost four times higher than that in the intensive treatment group.

Angiotensin-converting enzyme inhibitors (ACEIs) and angiotensin receptor blockers (ARBs), two of renoprotective renin–angiotensin–aldosterone system (RAAS) blockades, are correlated with acute kidney injury in critically ill patients[3]. According to commentary from the United States on the 2012 Kidney Disease: Improving Global Outcomes (KDIGO) guidelines[4], RAAS blockade remains the preferred drug for diabetic nephropathy with microalbuminuria. Non-dihydropyridine calcium channel blockers (CCBs) are recommended for hypertensive patients but not for those with CKD, according to the JNC 8 guidelines; however, a meta-analysis demonstrated that CCBs reduce not only BP but also proteinuria[5]. Therefore, Whether RAAS is more suitable than CCB for initial hypertension control in CKD patients is our study interest.

The present study conducted a systemic review and meta-analysis through a literature survey to elucidate whether RAAS blockade is still the most favorable therapeutic agent for hypertension treatment in patients with CKD. We included only RCTs that involved a direct head-to-head comparison between CCBs and the two RAAS blockades, ACEIs and ARBs, across different CKD stages and primary and secondary clinical measurement outcomes including the BP-lowering effect, mortality, heart failure, stroke or cerebrovascular, dialysis, renal function, and proteinuria.

## Materials and methods

The present systematic review and meta-analysis was conducted according to the Preferred Reporting Items for Systematic Reviews and Meta-Analyses (PRISMA) guidelines (S1 Table) [6]. This study was registered in PROSPERO with registration number CRD42017069375. Data are from the 21 randomized controlled trials whose authors' contact information can be found in the Supporting Information file S2 File.

### Search methods and eligibility criteria

The searches for relevant research articles that compared the effects of CCBs and two of RAAS blockades, ACEIs and ARBs, in patients with hypertension and CKD included the relative free-text and medical subject heading terms of chronic kidney disease, hypertension, calcium channel blockers, angiotensin-converting enzyme inhibitors, angiotensin II receptor antagonist, renin angiotensin aldosterone system in Cochrane Library, PubMed and Embase on 9^th^ November 2017 (S2 Table).

The obtained articles were screened by two different authors. They searched and reviewed the full text of all potentially eligible studies. The inclusion criteria was RCT that compared CCB and the two RAAS blockades in patients with hypertension and CKD. The exclusion criteria were as follows: renal transplantation, CKD stage 1 or 2, or combined therapy (data cannot be extracted separately). Any disagreement regarding article eligibility was resolved through discussions.

### Quality assessment for the included studies

The risk of bias in the included RCTs was assessed by two reviewers through the Cochrane risk of bias tool, and any disagreements were resolved by a third reviewer. Three aspects involving seven items associated with the risk of bias were evaluated: random sequence generation, allocation concealment, the blinding of participants and personnel, the blinding of assessment, incomplete outcome data, selective reporting, and other sources of bias.

### Data extraction and analysis

Two authors identified, double-checked, and extracted the data independently. They calculated MBP changes when articles provided relevant information on the baseline and endpoint values of SBP and DBP. Means and SDs were estimated from sample size, medians, and ranges when studies reported medians with minimum and maximum values[7]. Furthermore, when studies reported standard errors (SEs) instead of SDs, SDs were estimated based on the sample size (SE = SD/√N). Because risk ratio (RR) has some features which are better than odds ratio (OR), this systematic review and meta-analysis used RR rather than the OR for dichotomous data. RR can express outcome more intuitive than OR, and it was suggested to be used when the assumption of rare event can not be supported[8, 9]. The Peto OR was determined for dichotomous variables when a zero cell was present, and standard mean differences (SMDs) were applied for continuous variables. In addition, I^2^ values were used to estimate the heterogeneity among the included studies. A *P* value of <0.05 was considered statistically significant. The I^2^ was represented as the percentage of the total variability across the studies, and defined as 25%, 50%, and 75% for low, moderate, and high heterogeneity, respectively[10]. The results were expressed in forest plots conducted with RevMan version 5.3. The qualitative synthesis also assessed Egger’s regression intercept for publication bias. The subgroup analysis was according to CKD stage and baseline proteinuria.

## Results

**Fig 1:**
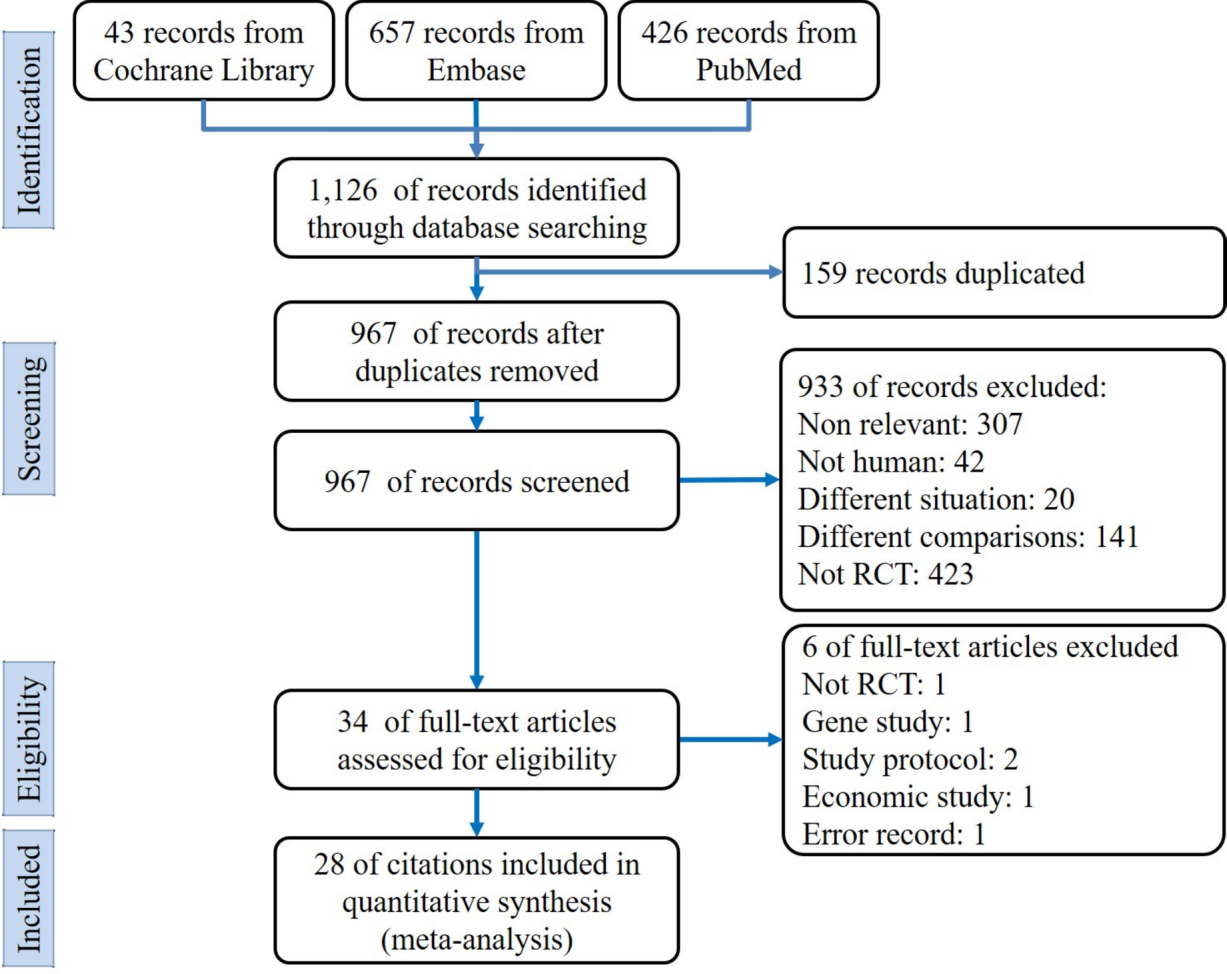
Flowchart of the systematic review and meta-analysis according to the PRISMA guidelines.

The search returned a total of 1126 (PubMed: 426, Embase: 657, and Cochrane Library: 43) citations, of which 159 were duplicates. According to the exclusion criteria, 933 citations were excluded after title and abstract screening. Because the citations were not RCTs, study protocols, gene studies, or economic studies, they were excluded in the full-text review phase. Fig 1 shows the literature survey and study selection processes.

### Characteristics of the included studies

The present systematic review included 21 RCTs that published as 28 journal articles [11–38]. These RCTs randomized 9492 patients with hypertension and advanced CKD into CCBs and the two RAAS blockades treatments. Table 1 lists the characteristics of the included studies, including location, years, patients, age, sex, CKD stages, blinding, and follow-up. All 21 eligible RCTs were published between 1992 and 2012. The recruitment period of these studies was approximately 14 years. Individual assessment of risk of bias was presented in S1 Fig.

**Table 1:** Characteristics and the risk of bias of the included studies.

**A**						
**Study**	**Location**		**Included years**		**No. of patients**	
					**CCB**	**ACEIs or ARBs**
AASK[11, 16, 35, 36]	United States		February 1995 to		217	436
			September 1998			
IDNT[12, 13, 23]	United States, Europe,		March 1996 to February		567	579
	Israel, Australasia, and		1999			
	Southeast Asia					
Campo et al.[14] (1997)	Barcelona, Spain		Unclear		11	13
Del Vecchio et al.[15]	Multicenter in Italy		Unclear		67	64
(2004)						
AVER[17]	Multicenter in Europe		Unclear		132	131
Fogari et al.[18] (1995)	Pavia, Italy		Unclear		20	20
Formica et al.[19]	United States		April 2001 to April 2003		27	29
(2006)						
Giri et al.[20] (2002)	New Delhi, India		Unclear		10	10
JLIGHT[21, 22]	Multicenter in Japan		December 1999 to March		59	58
			2002			
MacGregor et al.[24]	Multicenter in Scotland		June 1996 to January		28	28
(2005)			1999			
Marin et al.[25] (2001)	Multicenter in Spain		Unclear		112	129
Nakamura et al.[26]	Japan		Unclear		15	15
(2008)						
Petersen et al.[27]	Frederiksberg, Denmark		October 1992 to May		20	20
(2001)			1995			
Preston et al.[28] (1997)	United States		Unclear		180	187
ALLHAT-I[30, 31]	Multicenter in the United		February 1994 to January		1516	1533
	States, Canada, and Puerto		1998			
	Rico					
ALLHAT-II[29]	Multicenter in the United		February 2002 to January		1479	1501
	States, Canada, and Puerto		2006			
	Rico					
Rose et al.[32] (2001)	Saitama, Japan		Unclear		10	10
Shibasaki et al.[33]	Osaka, Japan		November 1998 to April		10	10
(2002)			2000			
Shibasaki et al.[34]	Osaka, Japan		September 2000 to		13	13
(2005)			November 2002			
Yilmaz et al.[37] (2010)	Ankara, Turkey		2004 to 2006		47	45
Zucchelli et al.[38]	Multicenter in Italy		Unclear		61	60
(1992)						
**B**						
**Study**	**Age**		**Sex (Female)**		**CKD stages**	
	**CCBs**	**orACEIs or ARBs**	**CCBs**	**ACEIs or ARBs**		
AASK[11, 16, 35, 36]	54.4 ± 10.7	54.2 ± 10.9	40.1%	38.8%	3 or 4	
IDNT[12, 13, 23]	59.1 ± 7.9	59.3 ± 7.1	37%	35%	Undefined	
Campo et al.[14]	60.6 ± 7.4	58.5 ± 11.1	36.4%	30.8%	3	
(1997)						
Del Vecchio et al.[15]	52.9 ± 10.5	56.4 ± 10.0	27%	34%	3 or 4	
(2004)						
AVER[17]	58.3 ± 11.3	57.5 ± 12.9	40.9%	40.5%	3 or 4	
Fogari et al.[18] (1995)	56.9 ± 1.3	57.1 ± 1.3	0%	0%	3	
Formica et al.[19]	46.9 ± 10.0	49 ± 11.2	44.4%	34.5%	ESRD	
(2006)						
Giri et al.[20] (2002)	Unclear	Unclear	Unclear	Unclear	Undefined	
JLIGHT[21, 22]	57.5 ± 11.9	55.7 ± 13.6	30.5%	37.9%	Undefined	
MacGregor et al.[24]	50	50	39%	54%	Undefined	
(2005)						
Marin et al.[25] (2001)	56 ± 14	53 ± 14	42.9%	38.8%	Undefined	
Nakamura et al.[26]	47 ± 10	45 ± 11	40%	40%	Undefined	
(2008)						
Petersen et al.[27]	54 ± 14	62 ± 9	35%	30%	Undefined	
(2001)						
Preston et al.[28]	Unclear	Unclear	0%	0%	Undefined	
(1997)						
ALLHAT-I[30, 31]	70.8 ± 7.6	70.6 ± 7.9	54.7%	49.8%	3 or 4	
ALLHAT-II[29]	70.8 ± 7.6	70.6 ± 7.9	54.8%	49.8%	3 or 4	
Rose et al.[32] (2001)	62.5 ± 3.6	62.4 ± 4.2	40%	40%	Undefined	
Shibasaki et al.[33]	56.4 ± 5.1	53.9 ± 4.1	40%	30% (ACEI)	ESRD	
(2002)		(ACEI)		40% (ARB)		
		54.2 ± 5.2 (ARB)				
Shibasaki et al.[34]	56.2 ± 3.9	56.4 ± 3.4	53.8%	38.5% (ACEI)	ESRD	
(2005)		(ACEI)		46.1% (ARB)		
		57.5 ± 4.4 (ARB)				
Yilmaz et al.[37]	49.2 ± 13.4	53.8 ± 17.6	42.6%	44.4%	ESRD	
(2010)						
Zucchelli et al.[38]	55 ± 10	55 ± 10	41.0%	43.3%	Undefined	
(1992)						
**C**						
**Study**	**Blinding**	**Follow-up**	**Loss to follow-up**		**Diabetes mellitus**	
AASK[11, 16, 35, 36]	Double-blind	36 months	11.1%		Excluded	
IDNT[12, 13, 23]	Double-blind	31 months	0.6%		Type 2 diabetes	
					mellitus were	
					included	
Campo et al.[14] (1997)	Open-label	4 weeks	0%		Unclear	
Del Vecchio et al.[15]	Double-blind	48 weeks	0%		Excluded	
(2004)						
AVER[17]	Double-blind	2.9 years	1.9%		Excluded	
Fogari et al.[18] (1995)	Double-blind	6 months	0%		Noninsulin-dependent	
					diabetes mellitus as	
					inclusion criteria	
Formica et al.[19]	Open-label	12 months	0%		44.6%	
(2006)						
Giri et al.[20] (2002)	Unclear	9 months	6.7%		Unclear	
JLIGHT[21, 22]	Open-label	12 months	Unclear		12.0%	
MacGregor et al.[24]	Open-label	4 years	Unclear		Excluded	
(2005)						
Marin et al.[25] (2001)	Open-label	3 years	Unclear		Excluded	
Nakamura et al.[26]	Double-blind	12 months	0%		Excluded	
(2008)						
Petersen et al.[27]	Double-blind	3.5 months	0%		20%	
(2001)						
Preston et al.[28]	Double-blind	6 to 9 years	Unclear		Unclear	
(1997)						
ALLHAT-I[30, 31]	Double-blind	4.9 years	2.3%		36%	
ALLHAT-II[29]	Double-blind	8.8 years	2.3%		36%	
Rose et al.[32] (2001)	Unclear	12 months	0%		Unclear	
Shibasaki et al.[33]	Double-blind	6 months	0%		40%	
(2002)						
Shibasaki et al.[34]	Double-blind	6 months	0%		38%	
(2005)						
Yilmaz et al.[37] (2010)	Unclear	12 months	0%		Excluded	
Zucchelli et al.[38]	Unclear	36 months	0%		Excluded	
(1992)						

### BP changes

**Fig 2:**
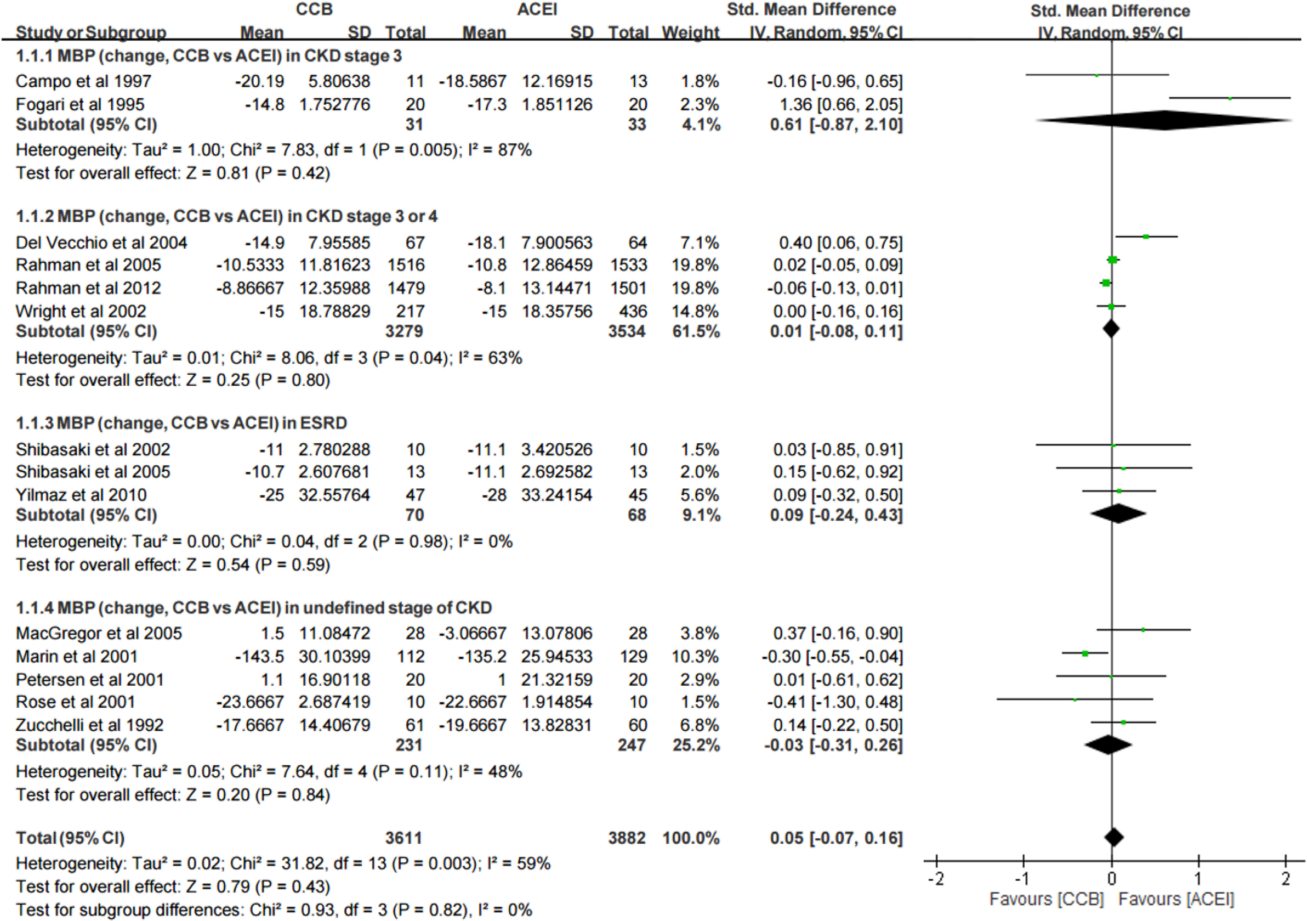
Forest plot of the meta-analysis for MBP changes between CCB and ACEI groups.

In the 14 included studies[14, 15, 18, 24, 25, 27, 29, 30, 32–34, 36–38] with 7493 patients, no significant differences in MBP changes were observed between the CCB and angiotensin-converting enzyme inhibitor (ACEI) groups (SMD, 0.05; 95% CI, −0.07 to 0.16; I^2^: 59%) (Fig 2, total). In the subgroups with different CKD stages, no significant differences were observed between the CCB and ACEI groups in CKD stage 3, mixed CKD stages 3 and 4, end-stage renal disease (ESRD), and undefined CKD stage data, the SMD were 0.61 (95% CI, −0.87, 2.10), 0.01 (95% CI, −0.08, 0.11), 0.09 (95% CI, −0.24, 0.43), −0.03 (95% CI, −0.31, 0.26). However, the studies including patients with CKD stage 3 and mixed CKD stages 3 and 4 exhibited different heterogeneity compared with those including patients with ESRD. The studies including patients with CKD stage 3 and mixed CKD stages 3 and 4 exhibited high (I^2^: 87%) and moderate (I^2^: 63%) heterogeneity, respectively; however, ESRD studies had low heterogeneity (I^2^: 0%). Because this result was consistent across the included studies only in the subgroup of ESRD, the result in the subgroup of ESRD might be more meaningful than in other subgroup. The publication bias of this result that was detected by Egger’s test showed non-significant (Egger’s test: *t* = 1.26; p > 0.05) (S1 File).

**Fig 3:**
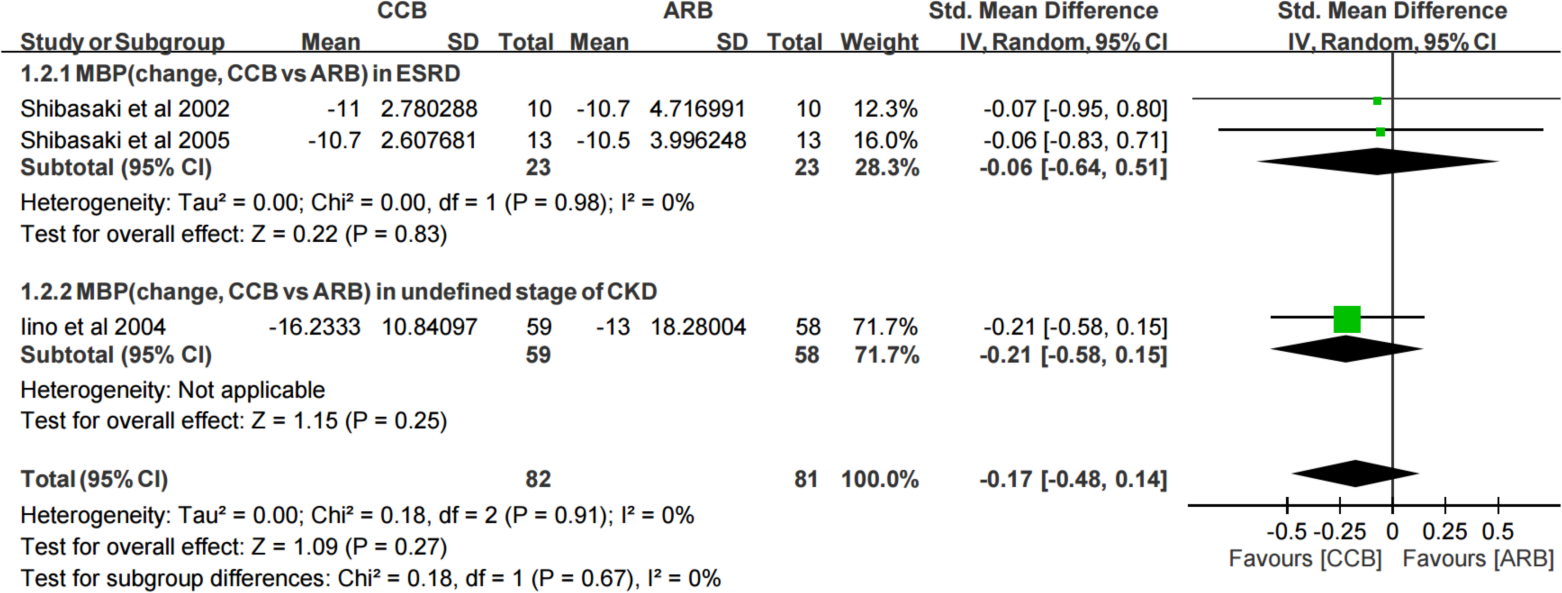
Forest plot of the meta-analysis for MBP changes between CCB and ARB groups.

In the three included studies[22, 33, 34] with 163 patients, no significant differences in MBP changes were observed between the CCB and angiotensin receptor blocker (ARB) groups (SMD, −0.17; 95% CI, −0.48, 0.14) (Fig 3, total). In the subgroups of ESRD and undefined CKD stage data, no differences in MBP were observed between two medications, the SMD were −0.06 (95% CI, −0.64, 0.51) and −0.21 (95% CI, −0.58, 0.15). Furthermore, these three studies had low heterogeneity (I^2^: 0%). The result was consistent across the included studies.

**Fig 4:**
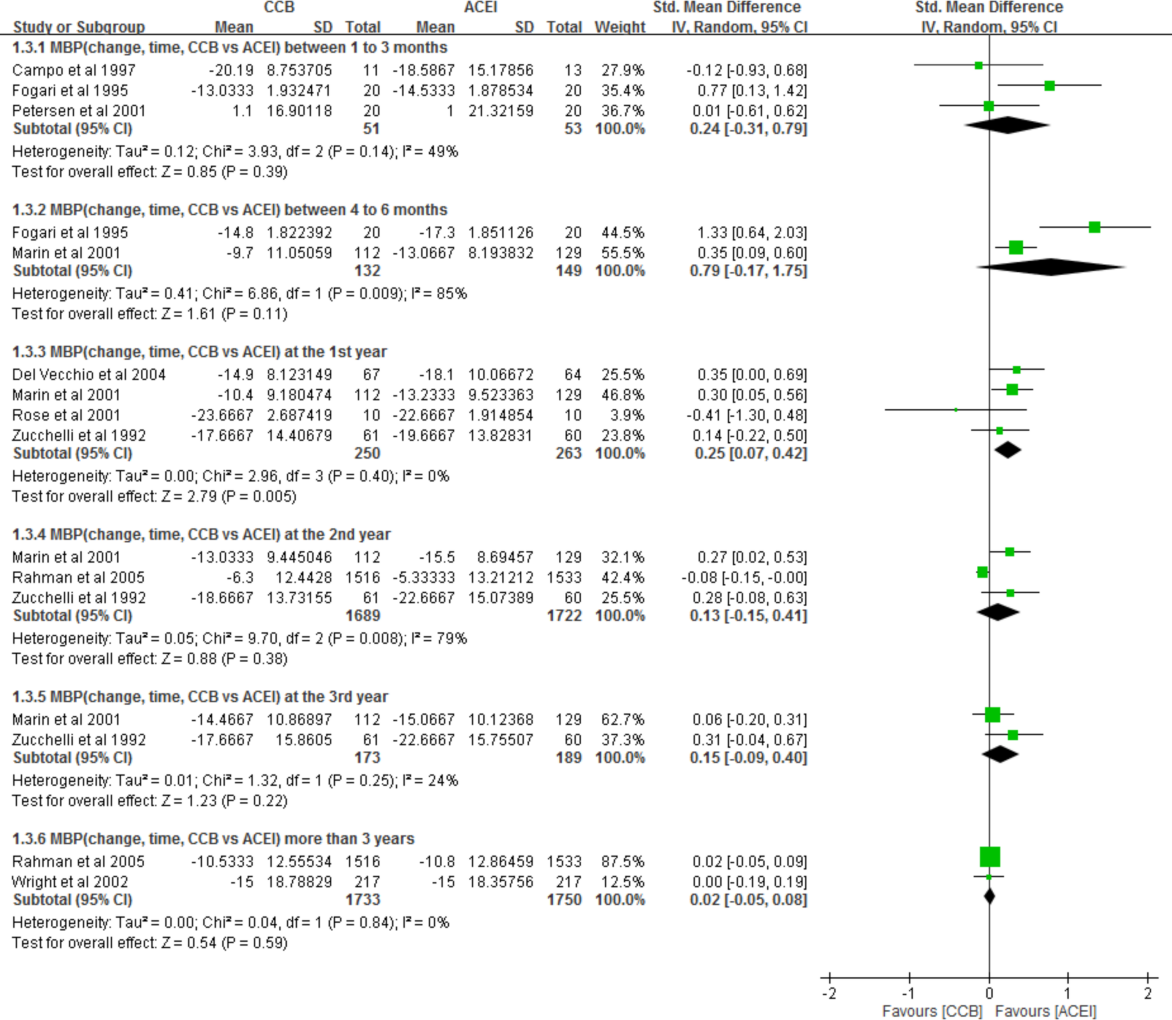
Forest plot of the meta-analysis for MBP changes between CCB and ACEI groups across different follow-up durations.

Nine studies[14, 15, 18, 25, 27, 30, 32, 36, 38] provided relevant information on MBP changes associated with CCBs and ACEIs and medication duration. The pooled data indicated that the ACEI group had a lower MBP at the first year (SMD, 0.25; 95% CI, 0.07, 0.42; p < 0.05) (Fig 4, 1.3.3); however, no significant differences were observed between ACEI and CCB groups at 1–3 months, 4–6 months, 2 years, 3 years, or more than 3 years, the SMD were 0.24 (95% CI, −0.31, 0.79), 0.79 (95% CI, −0.17, 1.75), 0.13 (95% CI, −0.15, 0.41), 0.15 (95% CI, −0.09, 0.40), and 0.02 (95% CI, −0.05, 0.08).

In the further analysis of blood pressure, the present meta-analysis analyzed changes of MBP, SBP, and DBP that stratified by CKD stage and baseline proteinuria in Table 2 (S2–S6 Figs). Ten studies[14, 15, 18, 24, 25, 27, 29, 30, 32, 36] reported relevant information on SBP changes but did not exhibit significant differences between the CCB and ACEI groups (SMD, 0.02; 95% CI, −0.13, 0.16). No significant differences in SBP between two medications were found in subgroups of data of CKD stage 3, mixed CKD stages 3 and 4, and undefined CKD stage, the SMD were 0.38 (95% CI, −0.54, 1.30), 0.02 (95% CI, −0.05, 0.08), and −0.44 (95% CI, −1.19, 0.31).

**Table 2:** Further blood pressure outcome summary.

Outcomes	Studies(n)	CCB(n)	ACEI(n)	Effect size	95% CI	I^2^
**SBP changes**^a^						
CKD stage 3	2	31	33	0.38	[-0.54–1.30]	88%
Mixed CKD stages 3 and 4	4	3279	3315	0.02	[-0.05–0.08]	33%
Undefined CKD stage	4	170	187	-0.44	[-1.19–0.31]	86%
Total	10	3480	3535	0.02	[-0.13–0.16]	75%
**DBP changes**^a^						
CKD stage 3	2	31	33	0.50	[-0.64–1.64]	79%
Mixed CKD stages 3 and 4	4	3279	3315	-0.02	[-0.10–0.05]	44%
Undefined CKD stage	5	180	197	0.19	[-0.15–0.54]	46%
Total	11	3490	3545	0.06	[-0.06–0.18]	62%
**MBP changes (Stratified by baseline proteinuria)**^a^						
Proteinuria 0 to 1 mg/day	3	59	61	0.54	[-0.27–1.34]	77%
Proteinuria 0 to 3 mg/day	2	284	500	0.17	[-0.22–0.56]	76%
Proteinuria 0 to > 3 mg/day	1	61	60	0.14	[-0.22–0.50]	NE
Proteinuria undefined	8	3207	3261	-0.03	[-0.10–0.03]	14%
Total	14	3611	3882	0.05	[-0.07–0.16]	59%
**SBP changes (Stratified by baseline proteinuria)**^a^						
Proteinuria 0 to 1 mg/day	3	59	61	0.41	[-0.06–0.88]	37%
Proteinuria 0 to 3 mg/day	2	284	281	0.14	[-0.26–0.54]	76%
Proteinuria undefined	5	3137	3193	-0.11	[-0.29–0.07]	83%
Total	10	3480	3535	0.02	[-0.13–0.16]	75%
**DBP changes (Stratified by baseline proteinuria)**^a^						
Proteinuria 0 to 1 mg/day	3	59	61	0.44	[-0.17–1.06]	62%
Proteinuria 0 to 3 mg/day	2	284	281	0.02	[-0.20–0.24]	31%
Proteinuria 0 to > 3 mg/day	1	10	10	-0.44	[-1.33–0.45]	NE
Proteinuria undefined	5	3137	3193	0.02	[-0.11–0.14]	67%
Total	11	3490	3545	0.06	[-0.06–0.18]	62%

^a^Standard mean difference.

NE: no estimate.

In the 11 included studies[14, 15, 18, 20, 24, 25, 27, 29, 30, 32, 36] with 7035 patients, similar DBP changes were observed between the CCB and ACEI groups (SMD, 0.06; 95% CI, −0.15, 0.18) (Table 2). In the subgroups of data from CKD stage 3, mixed CKD stages 3 and 4, and undefined CKD stage, no significant differences in DBP between two medications were found, the SMD were 0.50 (95% CI, −0.64, 1.64), −0.02 (95% CI, −0.10, 0.05), and 0.19 (95% CI, −0.15, 0.54).

According to available information, the subgroup of baseline proteinuria in the further analysis were 0 to 1000 mg/day, 0 to 3000 mg/day, 0 to > 3000 mg/day, and undefined baseline proteinuria. The further subgroup analysis of MBP change from 14 RCTs[14, 15, 18, 24, 25, 27, 29, 30, 32–34, 36–38] showed that no significant differences between CCB and ACEI groups in all subgroups, the SMDs were 0.54 (95% CI, −0.27, 1.34), 0.17 (95% CI, −0.22, 0.56), 0.14 (95% CI, −0.22, 0.50), and -0.03 (95% CI, −0.10, 0.03) (Table 2).

The evidence of SBP change reported in 10 RCTs[14, 15, 18, 24, 25, 27, 29, 30, 32, 36] showed no significant differences between the two groups in subgroup of baseline proteinuria 0 to 1000 mg/day (SMD, 0.41; 95% CI, −0.06, 0.88), 0 to 3000 mg/day (SMD, 0.14; 95% CI, −0.26, 0.54), and undefined baseline proteinuria (SMD, -0.11; 95% CI, -0.29, 0.07) (Table 2).

The pooled data of DBP change presented in the 11 included RCTs[14, 15, 18, 20, 24, 25, 27, 29, 30, 32, 36] revealed that no significant differences between CCB and ACEI groups in all subgroups of baseline proteinuria 0 to 1000 mg/day (SMD, 0.44; 95% CI, -0.17, 1.06), 0 to 3000 mg/day (SMD, 0.02; 95% CI, -0.20, 0.24), 0 to > 3000 mg/day (SMD, -0.44; 95% CI, -1.33, 0.45), and undefined baseline proteinuria (SMD, 0.02; 95% CI, -0.11, 0.14) (Table 2).

### Mortality, heart failure, stroke, and renal outcomes

In Table 3, seven included studies[11, 17, 23–25, 29, 38] with 5460 patients provided relevant information on mortality and revealed no significant differences between the CCB and ACEI groups (n = 884 [34.05%] vs n = 869 [30.34%]; Peto OR, 1.09; 95% CI, 0.96, 1.24) (S7 Fig). The subgroups of mixed CKD stages 3 and 4, and undefined CKD stage showed no differences in mortality between two medications, the Peto OR were 1.11 (95% CI, 0.96, 1.28) and 0.98 (95% CI, 0.72, 1.34). There were 1897 patients dead from cardiovascular disease (CVD) among the 5219 patients in 5 RCTs of the 6 RCTs. However, no evidence showed significant differences in CVD mortality rate between CCB and ACEI groups in the all of CKD stage subgroups (S8 Fig). In the subgroup of mixed CKD stages 3 and 4, there were 903 CVD mortalities among 3212 patients in CCB group and 902 CVD mortalities among 3470 in ACEI group, the RR was 1.01 (95% CI, 0.94, 1.09). In subgroup of undefined CKD stage, pooled results from 2 RCTs revealed that CCB group has similar CVD mortality rate to ACEI group (n = 38 [5.60%] vs n = 54 [7.63%]), the RR was 0.72 (95% CI, 0.48, 1.08).

**Table 3:** Mortality and renal outcome summary.

Outcomes	Studies(n)	CCB(n)	ACEI(n)	Effect size	95% CI	I^2^
**Mortality**^a^						
Mixed CKD stages 3 and 4	3	792/1828	773/2068	1.11	[0.96–1.28]	0%
Undefined CKD stage	4	92/768	96/796	0.98	[0.72–1.34]	0%
Total	7	884/2596	869/2864	1.09	[0.96–1.24]	0%
**CVD mortality**^b^						
Mixed CKD stages 3 and 4	3	903/3212	902/3470	1.01	[0.94–1.09]	0%
Undefined CKD stage	2	38/679	54/708	0.72	[0.48–1.08]	0%
Total	5	941/3891	956/4178	1.00	[0.93–1.08]	0%
**Heart failure**^b^						
Mixed CKD stages 3 and 4	2	378/2995	385/3034	0.99	[0.86–1.14]	9%
Undefined CKD stage	1	93/567	60/579	1.58	[1.17–2.14]	NE
Total	3	471/3562	446/3613	1.13	[0.87–1.47]	77%
**Stroke/CVA**^b^						
Mixed CKD stages 3 and 4	2	165/2995	158/3034	1.06	[0.86–1.31]	0%
Undefined CKD stage	2	17/679	29/708	0.69	[0.24–1.98]	24%
Total	4	182/3674	187/3742	0.96	[0.72–1.28]	34%
**Dialysis**^b^						
Mixed CKD stages 3 and 4	3	217/2575	235/2765	1.02	[0.84–1.26]	22%
Undefined CKD stage	3	167/656	207/667	1.25	[1.00–1.56]	6%
Total	6	354/3231	342/3432	1.12	[0.95–1.31]	25%
**GFR**^b^						
Mixed CKD stages 3 and 4	2	57/1733	80/1969	1.03	[0.62–1.72]	59%
Undefined CKD stage	2	127/595	109/607	1.19	[0.95–1.49]	0%
Total	4	184/2328	189/2576	1.14	[0.95–1.37]	0%
**Urinary protein excretion changes**^c^						
Mixed CKD stages 3 and 4	1	67	64	0.17	[-0.17–0.51]	NE
Undefined CKD stage	2	71	70	0.83	[-0.82–2.47]	88%
Total	3	138	134	0.84	[0.65–1.15]	77%

^a^Peto odds ratio.

^b^Risk ratio.

^c^Standard mean difference.

NE: no estimate. CKD: chronic kidney disease. CVD: cardiovascular disease. CVA: cerebrovascular accident. GFR: glomerular filtration rate.

The quantitative synthesis of the included 3 RCTs[13, 29, 31] showed CCBs has similar heart failure rate to ACEIs in overall (n = 471 [13.22%] vs n = 464 [12.34%]), the RR was 1.13 (95% CI, 0.87, 1.47) (S9 Fig). The results of heart failure rate in subgroup analysis showed that no difference between CCBs and ACEIs in subgroup of mixed CKD stages 3 and 4, the RR was 0.99 (95% CI, 0.86, 1.14); but significant difference in heart failure rate between CCBs and ACEIs was found in subgroup of undefined CKD stage, the RR was 1.58 (95% CI, 1.17, 2.14). Because the evidence on heart failure rate between in the subgroup of undefined CKD stage was based on only one RCT, this result might not provide well conclusion on this issue.

Four of the included RCTs[13, 25, 29, 31] reported information of stroke or cerebrovascular accident (CVA). The results of meta-analysis showed no significant differences in stroke or CVA events between CCB and ACEI groups in both total (RR, 0.96; 95% CI, 0.72, 1.28) and subgroup analyses (S10 Fig). In subgroup of mixed CKD stages 3 and 4, CCBs has similar stroke or CVA events to ACEIs, the RR was 1.06 (95% CI, 0.86, 1.31). In subgroup of undefined CKD stage, no evidence exhibited difference in stroke or CVA events between the two medication groups, the RR was 0.69 (95% CI, 0.24, 1.98).

Six included studies[11, 23, 24, 29, 30, 38] with 6663 patients provided relevant information on dialysis events associated with CCBs and ACEIs. These studies did not exhibit significant differences in dialysis events between the CCB and ACEI groups (n = 354 [9.38%] vs n = 342 [9.11%]; RR, 1.12; 95% CI, 0.95, 1.31). In the subgroups with different CKD stages, no significant differences were observed between the CCB and ACEI groups in mixed CKD stages 3 and 4 data (RR, 1.02; 95% CI, 0.84, 1.26) but significant results were only found in undefined CKD stage data (RR, 1.25; 95% CI, 1.00, 1.56], p = 0.05) (Table 3) (S11 Fig).

In the meta-analysis of the glomerular filtration rate (GFRs), four studies[11, 23, 24, 30] with 4904 patients showed no significant differences in the GFRs between the CCB and ACEI groups (n = 184 [7.90] vs n = 189 [7.34%]; RR, 1.14; 95% CI, 0.95, 1.37). In the subgroups with different CKD stages, no significant differences were observed between the CCB and ACEI groups in mixed CKD stages 3 and 4 (RR, 1.03; 95% CI, 0.62, 1.72) and undefined CKD stage (RR, 1.19; 95% CI, 0.95, 1.49) data (Table 3) (S12 Fig).

In the meta-analysis of urinary protein excretion changes, three studies[15, 20, 38] with 272 patients showed no significant differences in urinary protein excretion changes between the CCB and ACEI groups (SMD, 0.42; 95% CI, −0.17, 1.00). In the subgroups with different CKD stages, no significant differences were observed between the CCB and ACEI groups in mixed CKD stages 3 and 4 (SMD, 0.17; 95% CI, −0.17, 0.51) and undefined CKD stage (SMD, 0.83; 95% CI, −0.82, 2.47) data (Table 3) (S13 Fig).

## Discussion

### CCBs is not inferior to ACEIs and ARBs in CKD stage 3 to 5D

The present study demonstrates similar therapeutic effects between groups of CCB and the two RAAS blockades, ACEIs and ARBs, with regard to BP-lowering effects, such as changes in MBP, SBP, and DBP, in hypertensive patients with CKD stage 3, mixed CKD stages 3 and 4, and ESRD who received renal replacement therapy. The changes in subgroup analysis for MBP across different follow-up durations reveal that the ACEI group achieved improved outcomes compared with the CCB group only in the first year of follow-up but did not exhibit significant differences in long-term follow-up, suggesting the escape phenomenon of ACEIs. Generally, no difference in the efficacy of the BP-lowering effects between CCBs and ACEIs.

No significant differences were observed between the CCB and ACEI groups with regard to mortality, as well as CVD mortality. Furthermore, the two groups did not exhibit significant differences with regard to stroke or CVA events, dialysis events, GFRs, and urinary protein excretion changes, suggesting that CCBs also exert renal protection effects and are not inferior to ACEIs in preventing the loss of renal function.

### Mechanism of the two RAAS blockades and CCBs

The RAAS plays a pivotal role in the pathogenesis of hypertension. Angiotensinogen, released by the liver, is converted to angiotensin I by renin, an enzyme synthesized by the juxtaglomerular cells in the renal glomerulus. Angiotensin I is then cleaved by ACE to angiotensin II, which acts as the primary active product of the RAAS. Angiotensin II primarily acts through the angiotensin-1 (AT-1) receptor to promote systemic arterial vasoconstriction, renal arteriolar vasoconstriction, the stimulation of the renal tubular reabsorption of sodium and water, and aldosterone release from the adrenal glands, leading to elevated SBP and DBP. RAAS dysregulation is involved in the pathogenesis of several hypertensive disorders[39].

RAAS blockade has been shown to be beneficial in patients with hypertension. ACEIs and ARBs are the major RAAS inhibitors commonly used in clinical practice. ACEIs competitively block the action of ACE, preventing the conversion of angiotensin I to angiotensin II. ACEIs can also reduce aldosterone and vasopressin secretion and sympathetic nerve activity[40]. Furthermore, ACEIs prevent the breakdown of bradykinin, which increases the production of nitric oxide and results in vasodilatation[41]. These effects of sodium and water excretion and vasodilatation contribute to the effective reduction of SBP and DBP. However, chronic ACEI use may be associated with the reduction of angiotensin II and aldosterone concentrations to baseline levels despite ACE blockage, which is referred to as the ACE escape phenomenon and limits the efficacy of ACEIs[42]. Moreover, the elevation of bradykinin concentration is implicated with unpleasant side effects, such as cough and angioedema[43]. ARBs specifically prevent the binding of angiotensin II to AT-1 receptors but not AT-2 receptors, thus inhibiting all deleterious effects modulated by angiotensin II but preserving the potential beneficial effects of the AT-2 receptor pathway as well as ensuring that the BP-lowering effects of ARBs are as effective as those of ACEIs. Most adverse events related to ACEI and ARB therapies have been associated with the potential effects of RAAS blockade, including hypotension, hyperkalemia, and aggravated renal function. Both ACEs and ARBs are contraindicated in pregnant women because RAAS blockade is associated with increased fetal morbidity and mortality.

CCBs inhibit the flow of extracellular calcium through voltage-gated L-type calcium channels, which are responsible for the excitation of the smooth and cardiac muscles and aldosterone secretion from the adrenal cortex in humans. When calcium influx is inhibited, the vascular smooth muscle cells relax, resulting in vasodilation and BP reduction. CCBs also reduce the contractility of the cardiac muscles and decelerate sinus pacing and atrioventricular conduction[44]. Two major subtypes of CCBs bind to separate sites on the L-type calcium channel: dihydropyridines (e.g., nifedipine and amlodipine) and nondihydropyridines (e.g., diltiazem and verapamil)[45]. All CCBs are vasodilators that exert BP-lowering effects across all patient groups, regardless of sex, ethnicity, age, and dietary sodium intake. Nimodipine has been approved for short-term use in patients with subarachnoid hemorrhage but has not been indicated for hypertension treatment. Dihydropyridine CCBs are less likely to reduce cardiac output than nondihydropyridine CCBs because nondihydropyridine CCBs can exert negative inotropic effects. CCBs cause natriuresis by increasing renal blood flow, dilating the afferent arterioles, and increasing glomerular filtration pressure[46]. Nondihydropyridine CCBs exert substantial antialbuminuric effects compared with dihydropyridine CCBs by improving glomerular permselectivity and/or reducing the renal perfusion pressure. Dihydropyridine CCBs are highly heterogeneous with respect to antiproteinuric and renal protection effects, which can be attributed to the blockade of T receptors in the glomerular efferent arteriole[47]. Common adverse effects of CCBs include edema, flushing, headache, dizziness, and constipation.

### Strengths of the present study

Because the present systematic review included a large number of RCTs, subgroup analysis, and modified statistical methods, it could provide stronger evidence than previous studies as well as previous meta-analyses[48]. The previous meta-analysis reviewed 530 citations and included 10 RCTs to derive the conclusion that RAAS blockade is superior to CCBs in terms of ESRD events but has a similar effect on mortality, as demonstrated by forest plots without subgroup analysis.

In the present systematic review, 1126 citations were found, in which 28 citations were from 21 RCTs. All 10 RCTs included in the previous systematic review was also included in the present study. Although this study only focused on advanced CKD, the information and cases were more than previous study. The present study also conducted subgroup analyses for CKD stages with available data. The present meta-analysis used RR rather than OR for dichotomous data, because OR always overestimates effects[8, 49]. Moreover, this meta-analysis used Peto OR when a zero cell was present in the dichotomous data. Therefore, the present study may provide more reliable evidence.

### Limitations and directions for future studies

The present study has some limitations. First, we did not evaluate the dual or combination therapy of CCBs and RAAS blockade for severe hypertension. After the ACCOMPLISH trial based on ACEIs and add-on CCBs or diuretics design[50], monotherapy (either CCBs or RAAS blockade) remained the preferred treatment for mild to moderate hypertension. Second, we only conducted head to head comparison of CCBs and two RAAS blockades, ACEIs and ARBs, in the included RCTs. Evaluation of the direct or indirect effects of combined drug treatments is complex and therefore warrants additional network meta-analyses. Third, we did not categorize or stratify patients based on the presence or absence diabetes. Fourth, the heterogeneity across studies was found in the outcomes of heart failure, urinary protein excretion changes, and blood pressure expect the subgroup of ESRD. These limits might be dealt in future by well-designed and well-structured RCT or network meta-analysis.

## Conclusions

Hypertension is one of the most common presentations and is highly associated with morbidity and mortality in patients with CKD. However, uniform agreement has not been established regarding the type of antihypertensive drugs to be used for initial therapy. CCBs and two RAAS blockades, ACEIs and ARBs, are highly potent agents that have been frequently used as initial antihypertensive agents for mild to moderate hypertension. In contrast to the current KDIGO guideline that recommends RAAS blockade as the first-line therapy in nondiabetic and proteinuric patients with CKD[1, 4], the present study found no evidence of absence of the examined effects on long-term BP, mortality, heart failure, stroke or CVA events, renal function, and urinary protein levels in patients with CKD, irrespective of the presence or absence of diabetes. Our study results are in accordance with the 2007 American Heart Association and 2013 European Society of Hypertension guidelines[51, 52], which state that the major determinant is lower antihypertensive agents but not the choice of antihypertensive agents. Because the heterogeneity across studies was found in some results, the conclusion should be warranted by well-designed studies and further discussions in future.

## Supporting information

S1 Table PRISMA checklist. (PDF)

S2 Table Search strategy. (PDF)

S1 File Publication bias analysis. (PDF)

S2 File Author contact information. (PDF)

S1 Fig Risk of bias summary. (TIF)

S2 Fig Forest plot of the meta-analysis for SBP changes. (TIF)

S3 Fig Forest plot of the meta-analysis for DBP changes. (TIF)

S4 Fig Forest plot of the meta-analysis for MBP changes (Subgroups of baseline proteinuria). (TIF)

S5 Fig Forest plot of the meta-analysis for SBP changes (Subgroups of baseline proteinuria). (TIF)

S6 Fig Forest plot of the meta-analysis for DBP changes (Subgroups of baseline proteinuria). (TIF)

S7 Fig Forest plot of the meta-analysis for mortality. (TIF)

S8 Fig Forest plot of the meta-analysis for CVD mortality. (TIF)

S9 Fig Forest plot of the meta-analysis for heart failure. (TIF)

S10 Fig Forest plot of the meta-analysis for stroke or CVA events. (TIF)

S11 Fig Forest plot of the meta-analysis for dialysis. (TIF)

S12 Fig Forest plot of the meta-analysis for GFR events. (TIF)

S13 Fig Forest plot of the meta-analysis for urinary protein excretion. (TIF)

## References

[1] James PA, Oparil S, Carter BL, Cushman WC, Dennison-Himmelfarb C, Handler J, et al 2014 evidence-based guideline for the management of high blood pressure in adults: report from the panel members appointed to the Eighth Joint National Committee (JNC 8). Jama. 2014;311(5):507–20. Epub 2013/12/20. doi: 10.1001/jama.2013.284427 . 24352797 10.1001/jama.2013.284427

[2] Wright JT Jr, Williamson JD, Whelton PK, Snyder JK, Sink KM, Rocco MV, et al A Randomized Trial of Intensive versus Standard Blood-Pressure Control. The New England journal of medicine. 2015;373(22):2103–16. Epub 2015/11/10. doi: 10.1056/NEJMoa1511939 ; PubMed Central PMCID: PMCPMC4689591. 26551272 10.1056/NEJMoa1511939 PMC4689591

[3] Lim HJ, Lee HH, Kim AJ, Ro H, Kim HS, Chang JH, et al Renin-Angiotensin-Aldosterone System Blockade in Critically Ill Patients Is Associated with Increased Risk for Acute Kidney Injury. The Tohoku journal of experimental medicine. 2016;238(1):17–23. Epub 2015/12/15. doi: 10.1620/tjem.238.17 . 26656621 10.1620/tjem.238.17

[4] Taler SJ, Agarwal R, Bakris GL, Flynn JT, Nilsson PM, Rahman M, et al KDOQI US commentary on the 2012 KDIGO clinical practice guideline for management of blood pressure in CKD. American journal of kidney diseases: the official journal of the National Kidney Foundation. 2013;62(2):201–13. Epub 2013/05/21. doi: 10.1053/j.ajkd.2013.03.018 ; PubMed Central PMCID: PMCPMC3929429. 23684145 10.1053/j.ajkd.2013.03.018 PMC3929429

[5] Thamcharoen N, Susantitaphong P, Wongrakpanich S, Chongsathidkiet P, Tantrachoti P, Pitukweerakul S, et al Effect of N- and T-type calcium channel blocker on proteinuria, blood pressure and kidney function in hypertensive patients: a meta-analysis. Hypertension research: official journal of the Japanese Society of Hypertension. 2015;38(12):847–55. Epub 2015/07/03. doi: 10.1038/hr.2015.69 . 26134125 10.1038/hr.2015.69

[6] Moher D, Liberati A, Tetzlaff J, Altman DG. Preferred reporting items for systematic reviews and meta-analyses: the PRISMA statement. PLoS medicine. 2009;6(7):e1000097 Epub 2009/07/22. doi: 10.1371/journal.pmed.1000097 ; PubMed Central PMCID: PMCPMC2707599. 19621072 10.1371/journal.pmed.1000097 PMC2707599

[7] Hozo SP, Djulbegovic B, Hozo I. Estimating the mean and variance from the median, range, and the size of a sample. BMC medical research methodology. 2005;5:13 Epub 2005/04/21. doi: 10.1186/1471-2288-5-13 ; PubMed Central PMCID: PMCPMC1097734. 15840177 10.1186/1471-2288-5-13 PMC1097734

[8] Deeks JJ. Issues in the selection of a summary statistic for meta-analysis of clinical trials with binary outcomes. Stat Med. 2002;21(11):1575–600. doi: 10.1002/sim.1188 . 12111921 10.1002/sim.1188

[9] Stare J, Maucort-Boulch D. Odds Ratio, Hazard Ratio and Relative Risk. Metodoloski Zvezki. 2016;13(1):59.

[10] Higgins JP, Thompson SG, Deeks JJ, Altman DG. Measuring inconsistency in meta-analyses. BMJ. 2003;327(7414):557–60. doi: 10.1136/bmj.327.7414.557 ; PubMed Central PMCID: PMCPMC192859. 12958120 10.1136/bmj.327.7414.557 PMC192859

[11] Agodoa LY, Appel L, Bakris GL, Beck G, Bourgoignie J, Briggs JP, et al Effect of ramipril vs amlodipine on renal outcomes in hypertensive nephrosclerosis: a randomized controlled trial. Jama. 2001;285(21):2719–28. Epub 2001/06/21. . 11386927 10.1001/jama.285.21.2719

[12] Atkins RC, Briganti EM, Lewis JB, Hunsicker LG, Braden G, Champion de Crespigny PJ, et al Proteinuria reduction and progression to renal failure in patients with type 2 diabetes mellitus and overt nephropathy. American journal of kidney diseases: the official journal of the National Kidney Foundation. 2005;45(2):281–7. Epub 2005/02/03. . 15685505 10.1053/j.ajkd.2004.10.019

[13] Berl T, Hunsicker LG, Lewis JB, Pfeffer MA, Porush JG, Rouleau JL, et al Cardiovascular outcomes in the Irbesartan Diabetic Nephropathy Trial of patients with type 2 diabetes and overt nephropathy. Annals of internal medicine. 2003;138(7):542–9. Epub 2003/04/02. . 12667024 10.7326/0003-4819-138-7-200304010-00010

[14] Campo C, Garcia-Vallejo O, Barrios V, Lahera V, Manero M, Esteban E, et al The natriuretic effect of nifedipine gastrointestinal therapeutic system remains despite the presence of mild-to moderate renal failure. Journal of Hypertension. 1997;15(12 II):1803–8. 9488243 10.1097/00004872-199715120-00093

[15] Del Vecchio L, Pozzi M, Salvetti A, Maschio G, Fusaroli M, Rovati C, et al Efficacy and tolerability of manidipine in the treatment of hypertension in patients with non-diabetic chronic kidney disease without glomerular disease. Prospective, randomized, double-blind study of parallel groups in comparison with enalapril. Journal of Nephrology. 2004;17(2):261–9. 15293527

[16] Douglas JG, Agodoa L. ACE inhibition is effective and renoprotective in hypertensive nephrosclerosis: the African American Study of Kidney Disease and Hypertension (AASK) trial. Kidney international Supplement. 2003;(83):S74–6. Epub 2003/07/17. doi: 10.1046/j.1523-1755.63.s83.15.x . 12864879 10.1046/j.1523-1755.63.s83.15.x

[17] Esnault VLM, Brown EA, Apetrei E, Bagon J, Calvo C, DeChatel R, et al The effects of amlodipine and enalapril on renal function in adults with hypertension and nondiabetic nephropathies: A 3-year, randomized, multicenter, double-blind, placebo-controlled study. Clinical Therapeutics. 2008;30(3):482–98. doi: 10.1016/j.clinthera.2008.03.006 18405787 10.1016/j.clinthera.2008.03.006

[18] Fogari R, Zoppi A, Pasotti C, Mugellini A, Lusardi P, Lazzari P, et al Comparative effects of ramipril and nitrendipine on albuminuria in hypertensive patients with non-insulin-dependent diabetes mellitus and impaired renal function. Journal of human hypertension. 1995;9(2):131–5. Epub 1995/02/01. . 7752175

[19] Formica RN Jr., Friedman AL, Lorber MI, Smith JD, Eisen T, Bia MJ. A randomized trial comparing losartan with amlodipine as initial therapy for hypertension in the early post-transplant period. Nephrology, dialysis, transplantation: official publication of the European Dialysis and Transplant Association—European Renal Association. 2006;21(5):1389–94. Epub 2006/01/25. doi: 10.1093/ndt/gfk058 . 16431893 10.1093/ndt/gfk058

[20] Giri S, Mahajan SK, Sen R, Sharma A. Effects of angiotensin converting enzyme inhibitor on renal function in patients of membranoproliferative glomerulonephritis with mild to moderate renal insufficiency. The Journal of the Association of Physicians of India. 2002;50:1245–9. Epub 2003/02/06. . 12568207

[21] Iino Y, Hayashi M, Kawamura T, Shiigai T, Tomino Y, Yamada K, et al Interim evidence of the renoprotective effect of the angiotensin II receptor antagonist losartan versus the calcium channel blocker amlodipine in patients with chronic kidney disease and hypertension: a report of the Japanese Losartan Therapy Intended for Global Renal Protection in Hypertensive Patients (JLIGHT) Study. Clinical and experimental nephrology. 2003;7(3):221–30. Epub 2003/10/31. doi: 10.1007/s10157-003-0241-3 . 14586719 10.1007/s10157-003-0241-3

[22] Iino Y, Hayashi M, Kawamura T, Shiigai T, Tomino Y, Yamada K, et al Renoprotective effect of losartan in comparison to amlodipine in patients with chronic kidney disease and hypertension—a report of the Japanese Losartan Therapy Intended for the Global Renal Protection in Hypertensive Patients (JLIGHT) study. Hypertension research: official journal of the Japanese Society of Hypertension. 2004;27(1):21–30. Epub 2004/04/02. . 15055252 10.1291/hypres.27.21

[23] Lewis EJ, Hunsicker LG, Clarke WR, Berl T, Pohl MA, Lewis JB, et al Renoprotective effect of the angiotensin-receptor antagonist irbesartan in patients with nephropathy due to type 2 diabetes. The New England journal of medicine. 2001;345(12):851–60. Epub 2001/09/22. doi: 10.1056/NEJMoa011303 . 11565517 10.1056/NEJMoa011303

[24] MacGregor MS, Deighan CJ, Rodger RS, Boulton-Jones JM. A prospective open-label randomised trial of quinapril and/or amlodipine in progressive non-diabetic renal failure. Nephron Clinical practice. 2005;101(3):c139–49. Epub 2005/07/15. doi: 10.1159/000086714 . 16015004 10.1159/000086714

[25] Marin R, Ruilope LM, Aljama P, Aranda P, Segura J, Diez J. A random comparison of fosinopril and nifedipine GITS in patients with primary renal disease. Journal of Hypertension. 2001;19(10):1871–6. 11593109 10.1097/00004872-200110000-00023

[26] Nakamura T, Inoue T, Suzuki T, Kawagoe Y, Ueda Y, Koide H, et al Comparison of renal and vascular protective effects between telmisartan and amlodipine in hypertensive patients with chronic kidney disease with mild renal insufficiency. Hypertension research: official journal of the Japanese Society of Hypertension. 2008;31(5):841–50. Epub 2008/08/21. doi: 10.1291/hypres.31.841 . 18712038 10.1291/hypres.31.841

[27] Petersen LJ, Petersen JR, Talleruphuus U, Moller ML, Ladefoged SD, Mehlsen J, et al A randomized and double-blind comparison of isradipine and spirapril as monotherapy and in combination on the decline in renal function in patients with chronic renal failure and hypertension. Clin Nephrol. 2001;55(5):375–83. Epub 2001/06/08. . 11393383

[28] Preston RA, Materson BJ, Reda DJ, Hamburger RJ, Williams DW, Smith MH. Proteinuria in mild to moderate hypertension: Results of the VA cooperative study of six antihypertensive agents and placebo. Clinical Nephrology. 1997;47(5):310–5. 9181278

[29] Rahman M, Ford CE, Cutler JA, Davis BR, Piller LB, Whelton PK, et al Long-term renal and cardiovascular outcomes in Antihypertensive and Lipid-Lowering Treatment to Prevent Heart Attack Trial (ALLHAT) participants by baseline estimated GFR. Clinical journal of the American Society of Nephrology: CJASN. 2012;7(6):989–1002. Epub 2012/04/12. doi: 10.2215/CJN.07800811 ; PubMed Central PMCID: PMCPMC3362309. 22490878 10.2215/CJN.07800811 PMC3362309

[30] Rahman M, Pressel S, Davis BR, Nwachuku C, Wright JT Jr., Whelton PK, et al Renal outcomes in high-risk hypertensive patients treated with an angiotensin-converting enzyme inhibitor or a calcium channel blocker vs a diuretic: a report from the Antihypertensive and Lipid-Lowering Treatment to Prevent Heart Attack Trial (ALLHAT). Arch Intern Med. 2005;165(8):936–46. Epub 2005/04/27. doi: 10.1001/archinte.165.8.936 . 15851647 10.1001/archinte.165.8.936

[31] Rahman M, Pressel S, Davis BR, Nwachuku C, Wright JT Jr., Whelton PK, et al Cardiovascular outcomes in high-risk hypertensive patients stratified by baseline glomerular filtration rate. Annals of internal medicine. 2006;144(3):172–80. Epub 2006/02/08. . 16461961 10.7326/0003-4819-144-3-200602070-00005

[32] Rose GW, Kanno Y, Ikebukuro H, Kaneko M, Kaneko K, Kanno T, et al Cilnidipine is as effective as benazepril for control of blood pressure and proteinuria in hypertensive patients with benign nephrosclerosis. Hypertension Research. 2001;24(4):377–83. 11510750 10.1291/hypres.24.377

[33] Shibasaki Y, Masaki H, Nishiue T, Nishikawa M, Matsubara H, Iwasaka T. Angiotensin II type 1 receptor antagonist, losartan, causes regression of left ventricular hypertrophy in end-stage renal disease. Nephron. 2002;90(3):256–61. Epub 2002/02/28. doi: 49060. doi: 10.1159/000049060 . 11867945 10.1159/000049060

[34] Shibasaki Y, Nishiue T, Masaki H, Tamura K, Matsumoto N, Mori Y, et al Impact of the angiotensin II receptor antagonist, losartan, on myocardial fibrosis in patients with end-stage renal disease: assessment by ultrasonic integrated backscatter and biochemical markers. Hypertension research: official journal of the Japanese Society of Hypertension. 2005;28(10):787–95. Epub 2006/02/14. doi: 10.1291/hypres.28.787 . 16471172 10.1291/hypres.28.787

[35] Weinberg JM, Appel LJ, Bakris G, Gassman JJ, Greene T, Kendrick CA, et al Risk of hyperkalemia in nondiabetic patients with chronic kidney disease receiving antihypertensive therapy. Arch Intern Med. 2009;169(17):1587–94. Epub 2009/09/30. doi: 10.1001/archinternmed.2009.284 . 19786678 10.1001/archinternmed.2009.284

[36] Wright JT Jr., Bakris G, Greene T, Agodoa LY, Appel LJ, Charleston J, et al Effect of blood pressure lowering and antihypertensive drug class on progression of hypertensive kidney disease: results from the AASK trial. Jama. 2002;288(19):2421–31. Epub 2002/11/21. . 12435255 10.1001/jama.288.19.2421

[37] Yilmaz R, Altun B, Kahraman S, Ozer N, Akinci D, Turgan C. Impact of amlodipine or ramipril treatment on left ventricular mass and carotid intima-media thickness in nondiabetic hemodialysis patients. Renal failure. 2010;32(8):903–12. Epub 2010/08/21. doi: 10.3109/0886022X.2010.502276 . 20722555 10.3109/0886022X.2010.502276

[38] Zucchelli P, Zuccala A, Borghi M, Fusaroli M, Sasdelli M, Stallone C, et al Long-term comparison between captopril and nifedipine in the progression of renal insufficiency. Kidney Int. 1992;42(2):452–8. Epub 1992/08/01. . 1405330 10.1038/ki.1992.309

[39] Laragh J. Laragh's lessons in pathophysiology and clinical pearls for treating hypertension. American journal of hypertension. 2001;14(2):186–94. Epub 2001/03/13. . 11243312 10.1016/s0895-7061(00)01317-0

[40] Lopez-Sendon J, Swedberg K, McMurray J, Tamargo J, Maggioni AP, Dargie H, et al Expert consensus document on angiotensin converting enzyme inhibitors in cardiovascular disease. The Task Force on ACE-inhibitors of the European Society of Cardiology. Eur Heart J. 2004;25(16):1454–70. Epub 2004/08/11. doi: 10.1016/j.ehj.2004.06.003 . 15302105 10.1016/j.ehj.2004.06.003

[41] Hornig B, Kohler C, Drexler H. Role of bradykinin in mediating vascular effects of angiotensin-converting enzyme inhibitors in humans. Circulation. 1997;95(5):1115–8. Epub 1997/03/04. . 9054837 10.1161/01.cir.95.5.1115

[42] Pitt B. "Escape" of aldosterone production in patients with left ventricular dysfunction treated with an angiotensin converting enzyme inhibitor: implications for therapy. Cardiovascular drugs and therapy. 1995;9(1):145–9. Epub 1995/02/01. . 7786835 10.1007/BF00877755

[43] Messerli FH, Nussberger J. Vasopeptidase inhibition and angio-oedema. Lancet (London, England). 2000;356(9230):608–9. Epub 2000/09/01. doi: 10.1016/s0140-6736(00)02596-4 . 10968427 10.1016/S0140-6736(00)02596-4

[44] Abernethy DR, Schwartz JB. Calcium-antagonist drugs. The New England journal of medicine. 1999;341(19):1447–57. Epub 1999/11/05. doi: 10.1056/NEJM199911043411907 . 10547409 10.1056/NEJM199911043411907

[45] Materson BJ. Calcium channel blockers. Is it time to split the lump? American journal of hypertension. 1995;8(3):325–9. Epub 1995/03/01. doi: 10.1016/0895-7061(94)00247-9 . 7794584 10.1016/0895-7061(94)00247-9

[46] Elliott WJ, Ram CV. Calcium channel blockers. J Clin Hypertens (Greenwich). 2011;13(9):687–9. Epub 2011/09/08. doi: 10.1111/j.1751-7176.2011.00513.x . 21896151 10.1111/j.1751-7176.2011.00513.x PMC8108866

[47] Hayashi K, Ozawa Y, Fujiwara K, Wakino S, Kumagai H, Saruta T. Role of actions of calcium antagonists on efferent arterioles—with special references to glomerular hypertension. American journal of nephrology. 2003;23(4):229–44. Epub 2003/07/04. doi: 72054. doi: 10.1159/000072054 . 12840599 10.1159/000072054

[48] Zhao HJ, Li Y, Liu SM, Sun XG, Li M, Hao Y, et al Effect of calcium channels blockers and inhibitors of the renin-angiotensin system on renal outcomes and mortality in patients suffering from chronic kidney disease: systematic review and meta-analysis. Renal failure. 2016;38(6):849–56. Epub 2016/04/09. doi: 10.3109/0886022X.2016.1165065 . 27055479 10.3109/0886022X.2016.1165065

[49] Schmidt CO, Kohlmann T. When to use the odds ratio or the relative risk? International journal of public health. 2008;53(3):165–7. 19127890 10.1007/s00038-008-7068-3

[50] Jamerson K, Weber MA, Bakris GL, Dahlof B, Pitt B, Shi V, et al Benazepril plus amlodipine or hydrochlorothiazide for hypertension in high-risk patients. The New England journal of medicine. 2008;359(23):2417–28. Epub 2008/12/05. doi: 10.1056/NEJMoa0806182 . 19052124 10.1056/NEJMoa0806182

[51] Mancia G, Fagard R. Have we attained the right control of hypertension? Consequences of the 2013 European Society of Hypertension/European Society of Cardiology and Eighth Joint National Committee recommendations. J Hypertens. 2014;32(9):1907 Epub 2014/08/08. doi: 10.1097/hjh.0000000000000277 . 25098788 10.1097/HJH.0000000000000277

[52] Rosendorff C. Hypertension and coronary artery disease: a summary of the American Heart Association scientific statement. J Clin Hypertens (Greenwich). 2007;9(10):790–5. Epub 2007/10/06. . 17917507 10.1111/j.1751-7176.2007.tb00006.x

