# Supplementary material for: Effects of calcium channel blockers comparing to angiotensin-converting enzyme inhibitors and angiotensin receptor blockers in patients with hypertension and chronic kidney disease stage 3 to 5 and dialysis: A systematic review and meta-analysis
Source: PLoS One. 2017 Dec 14;12(12):e0188975. doi: 10.1371/journal.pone.0188975 (PMC5730188; doi:10.1371/journal.pone.0188975)
Supplement: S2 Table — (PDF) [file pone.0188975.s002.pdf]

Search strategy

Database	Syntax	Returns
PubMed	(((("Renal Insufficiency"[Mesh] OR "Kidney Failure, Chronic"[Mesh] OR "chronic kidney failure" OR "chronic kidney insufficiency" OR "chronic renal failure" OR "chronic renal insufficiency" OR "kidney chronic failure" OR "kidney failure, chronic" OR "renal insufficiency, chronic")) AND ("Hypertension"[Mesh] OR "hypertension" OR "acute hypertension" OR "arterial hypertension" OR "blood pressure, high" OR "cardiovascular hypertension" OR "controlled hypertension" OR "endocrine hypertension" OR "high blood pressure" OR "high renin hypertension" OR "hypertensive disease" OR "hypertensive effect" OR "hypertensive response" OR "neurogenic hypertension" OR "preexistent hypertension" OR "refractory hypertension" OR "salt high blood pressure" OR "salt hypertension" OR "secondary hypertension" OR "systemic hypertension")) AND ("Calcium Channel Blockers"[Mesh] OR "calcium channel blocking agent" OR "calcium blocker" OR "calcium blocking agent" OR "calcium channel antagonist" OR "calcium channel blocker" OR "calcium channel blockers" OR "calcium channel blocking agent" OR "calcium entry blocker" OR "calcium entry blocking agent" OR "calcium inhibitor" OR ccb)) AND ("Angiotensin-Converting Enzyme Inhibitors"[Mesh] OR "Angiotensin Receptor Antagonists"[Mesh] OR "dipeptidyl carboxypeptidase inhibitor" OR "ace inhibitor" OR "angiotensin converting enzyme inhibiting agent" OR "angiotensin converting enzyme inhibitor" OR "angiotensin converting enzyme inhibitors" OR "angiotensin i converting enzyme inhibitor" OR "angiotensin-converting enzyme inhibitors" OR "converting enzyme inhibitor" OR "dipeptidyl carboxypeptidase i inhibitor" OR "dipeptidyl carboxypeptidase inhibitor" OR "kininase ii inhibitor" OR "peptidyl dipeptidase inhibitor" OR "peptidyldipeptide hydrolase inhibitor" OR arb OR "angiotensin receptor antagonist" OR "angiotensin ii receptor antagonist" OR "angiotensin ii receptor antagonists" OR "angiotensin ii receptor blocker" OR "angiotensin ii receptor blockers" OR "angiotensin ii receptor blocking agent" OR "angiotensin ii receptor blocking agents" OR "angiotensin receptor antagonist" OR "angiotensin receptor antagonists" OR "angiotensin receptor blocker" OR "angiotensin receptor blockers" OR "angiotensin receptor blocking agent" OR "angiotensin receptor blocking agents" OR "renin angiotensin aldosterone system" OR "angiotensin renin system" OR "kidney pressor system" OR "mechanism, renin angiotensin aldosterone" OR "renin aldosterone system" OR "renin angiotensin aldosterone mechanism" OR "renin angiotensin aldosterone system" OR "renin angiotensin mechanism" OR "renin angiotensin system" OR "renin-angiotensin system" OR "system, renin angiotensin" OR "system, renin angiotensin aldosterone")	426

Database	Syntax	Returns
Cochrane Library	#1. "Renal Insufficiency" OR "Kidney Failure, Chronic" OR "chronic kidney failure" OR "chronic kidney insufficiency" OR "chronic renal failure" OR "chronic renal insufficiency" OR "kidney chronic failure" OR "kidney failure, chronic" OR "renal insufficiency, chronic"<br>#2. "Hypertension" OR "hypertension" OR "acute hypertension" OR "arterial hypertension" OR "blood pressure, high" OR "cardiovascular hypertension" OR "controlled hypertension" OR "endocrine hypertension" OR "high blood pressure" OR "high renin hypertension" OR "hypertensive disease" OR "hypertensive effect" OR "hypertensive response" OR "neurogenic hypertension" OR "preexistent hypertension" OR "refractory hypertension" OR "salt high blood pressure" OR "salt hypertension" OR "secondary hypertension" OR "systemic hypertension"<br>#3. "Calcium Channel Blockers" OR "calcium channel blocking agent" OR "calcium blocker" OR "calcium blocking agent" OR "calcium channel antagonist" OR "calcium channel blocker" OR "calcium channel blockers" OR "calcium channel blocking agent" OR "calcium entry blocker" OR "calcium entry blocking agent" OR "calcium inhibitor" OR ccb<br>#4. "Angiotensin-Converting Enzyme Inhibitors" OR "Angiotensin Receptor Antagonists" OR "dipeptidyl carboxypeptidase inhibitor" OR "ace inhibitor" OR "angiotensin converting enzyme inhibiting agent" OR "angiotensin converting enzyme inhibitor" OR "angiotensin converting enzyme inhibitors" OR "angiotensin i converting enzyme inhibitor" OR "angiotensin-converting enzyme inhibitors" OR "converting enzyme inhibitor" OR "dipeptidyl carboxypeptidase i inhibitor" OR "dipeptidyl carboxypeptidase inhibitor" OR "kininase ii inhibitor" OR "peptidyl dipeptidase inhibitor" OR "peptidyldipeptide hydrolase inhibitor" OR arb OR "angiotensin receptor antagonist" OR "angiotensin ii receptor antagonist" OR "angiotensin ii receptor antagonists" OR "angiotensin ii receptor blocker" OR "angiotensin ii receptor blockers" OR "angiotensin ii receptor blocking agent" OR "angiotensin ii receptor blocking agents" OR "angiotensin receptor antagonist" OR "angiotensin receptor antagonists" OR "angiotensin receptor blocker" OR "angiotensin receptor blockers" OR "angiotensin receptor blocking agent" OR "angiotensin receptor blocking agents" OR "renin angiotensin aldosterone system" OR "angiotensin renin system" OR "kidney pressor system" OR "mechanism, renin angiotensin aldosterone" OR "renin aldosterone system" OR "renin angiotensin aldosterone mechanism" OR "renin angiotensin aldosterone system" OR "renin angiotensin mechanism" OR "renin angiotensin system" OR "renin-angiotensin system" OR "system, renin angiotensin" OR "system, renin angiotensin aldosterone"<br>#5. #1 AND #2 AND #3 AND #4	43
