# Supplementary material for: Effects of calcium channel blockers comparing to angiotensin-converting enzyme inhibitors and angiotensin receptor blockers in patients with hypertension and chronic kidney disease stage 3 to 5 and dialysis: A systematic review and meta-analysis
Source: PLoS One. 2017 Dec 14;12(12):e0188975. doi: 10.1371/journal.pone.0188975 (PMC5730188; doi:10.1371/journal.pone.0188975)
Supplement: S1 File — (PDF) [file pone.0188975.s003.pdf]

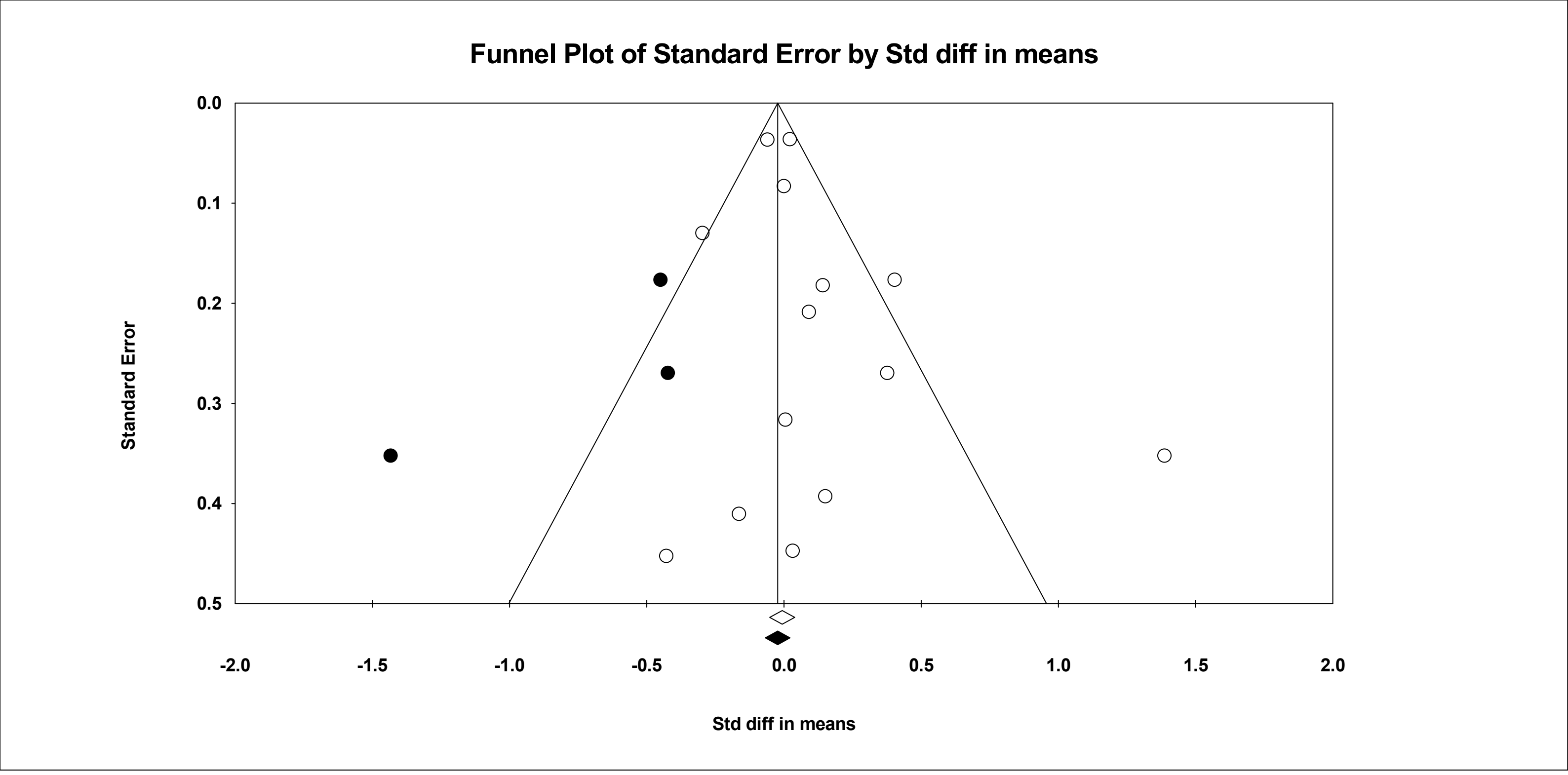

Funnel Plot of Standard Error by Std diff in means
Standard Error
0.0
0.1
0.2
0.3
0.4
0.5
-2.0
-1.5
-1.0
-0.5
0.0
0.5
1.0
1.5
2.0
Std diff in means

Begg and Mazumdar rank correlation

**Kendall's S statistic (P-Q)**	-9.00000

Kendall's tau without continuity correction

Tau	-0.09890
z-value for tau	0.49270
P-value (1-tailed)	0.31111
P-value (2-tailed)	0.62222

Kendall's tau with continuity correction

Tau	-0.08791
z-value for tau	0.43796
P-value (1-tailed)	0.33071
P-value (2-tailed)	0.66142

Egger's regression intercept

Intercept	0.71381
Standard error	0.56850
95% lower limit (2-tailed)	-0.52485
95% upper limit (2-tailed)	1.95246
t-value	1.25559
df	12.00000
P-value (1-tailed)	0.11658
P-value (2-tailed)	0.23316
