# Supplementary material for: Effects of calcium channel blockers comparing to angiotensin-converting enzyme inhibitors and angiotensin receptor blockers in patients with hypertension and chronic kidney disease stage 3 to 5 and dialysis: A systematic review and meta-analysis
Source: PLoS One. 2017 Dec 14;12(12):e0188975. doi: 10.1371/journal.pone.0188975 (PMC5730188; doi:10.1371/journal.pone.0188975)
Supplement: S2 File — (PDF) [file pone.0188975.s004.pdf]

Author Contact Information

Data are from the 21 randomized controlled trials whose authors' contact information are listed below:

1. Wright, Jr, MD, PhD, Case Western Reserve University, Clinical Hypertension Program, University Hospitals of Cleveland and the Louis Stokes Cleveland Veterans Affairs Medical Center, 10900 Euclid Ave, Wood Bldg Room W-165, Cleveland, OH 44106-4982 (e-mail: jxw20@po.cwru.edu)
2. Edmund J. Lewis, MD, Rush-Presbyterian-St. Luke's Medical Center, The Collaborative Study Group (CSG), 1750 West Harrison, Rawson Building, Room 522, Chicago, IL 60612 (e-mail: csg@rush.edu).
3. Carlos Campo Sien, Unidad de Hipertensión, Hospital 12 de Octubre, Ctra de Andalucía, Km 5.400, 28040 Madrid, Spain.
4. Prof. Francesco Locatelli, M.D. Department of Nephrology and Dialysis Ospedale Alessandro Manzoni Via dell'Eremo, 9, 23900 Lecco, Italy (e-mail: nefrologia@ospedale.lecco.it).
5. Vincent L.M. Esnault, MD, PhD, Néphrolo- gie, Pavillon S, Hôpital Pasteur, 30 voie Romaine, 06002 Nice, France (e-mail: esnault.v@chu-nice.fr)
6. Richard N. Formica Jr, MD, Department of Medicine/Section of Nephrology, FMP 106 P.O. Box 208029, New Haven, CT 06511–8029, USA. (e-mail: richard.formica@yale.edu)
7. Fogari R., Department of Internal Medicine and Therapeutics, University of Pavia, Italy.
8. Giri S., Department of Medicine, GTB Hospital and University College of Medical Sciences, Delhi - 110095, India.
9. Iino Y., Second Department of Medicine, Nippon Medical School, 1-1-5 Sendagi, Bunkyo-ku, Tokyo 113-8603, Japan Tel.: +81-3-3822-2131; Fax: +81-3-5802-8640 (email: iinoy@nms.ac.jp).
10. Dr. Mark S. MacGregor The John Stevenson Lynch Renal Unit Crosshouse Hospital, Kilmarnock KA2 0BE (UK) Tel.: +44 1563 577496, Fax: +44 1563 577493 (email: Mark.MacGregor@aaaht.scot.nhs.uk)
11. Rafael Marin, Servicio de Nefrologia, Hospital Covadonga, c/ Celestino Villamil s/n, 33006 Oviedo, Spain. Tel: +34 98 510 80 00; fax: +34 98 510 80 15 (e-mail: rmarini@senefro.org).
12. Teruo Inoue, M.D., Department of Cardiovascular and Renal Medicine, Saga University Faculty of Medicine, 5–1–1, Nabeshima, Saga 849–8501, Japan (e-mail: inouete@med.saga-u.ac.jp).

13. Dr. Petersen L. J., Department of Nephrology, Hvidovre Hospital, Denmark (e-mail: JPetersen@dadl.net).
14. Preston R.A., Hypertension Unit, Miami, FL 33136, United States.
15. Barry R. Davis, MD, PhD, University of Texas Health Science Center School of Public Health, 1200 Herman Pressler St, Suite E-801, Houston, TX 77030 (e-mail: barry.r.davis@uth.tmc.edu).
16. Dr. Charles E. Ford, The University of Texas School of Public Health, 1200 Herman Pressler Dr., Houston, Texas 77030 (e-mail: Charles.E.Ford@uth. tmc.edu)
17. Suzuki H., Department of Nephrology, Saitama Medical School, Iruma, Saitama 350-0495, Japan (e-mail: iromichi@saitama-med.ac.jp).
18. Hiroya Masaki, MD Department of Medicine II, Cardiovascular Center, Kansai Medical University 10–15 Fumizono-cho, Moriguchi Osaka 570-8507 (Japan) Fax +81 6 6998 6178 (e-mail: masakih@takii.kmu.ac.jp).
19. Yasunobu Shibasaki, M.D., Ph.D., Department of Medicine II, Kansai Medical University, Fumizono-cho 10−15, Moriguchi 570− 8507, Japan (e-mail: y_shibachan@ybb.ne.jp).
20. Rahmi Yilmaz, MD, Hacettepe Ü niversitesi Tip fakültesi Hastanesi Nefroloji Ü nitesi, 06100 Sihhiye, Ankara, Turkey (e-mail: drrahmiy@hotmail.com)
21. Prof. Pietro Zucche!li, Department of Nephrology, Ospedale M. Ma/pighi, Via P. Pa/agi, 9, 40138 Bologna, Italy.
