# Supplementary figures and images for: Effects of calcium channel blockers comparing to angiotensin-converting enzyme inhibitors and angiotensin receptor blockers in patients with hypertension and chronic kidney disease stage 3 to 5 and dialysis: A systematic review and meta-analysis
Source: PLoS One. 2017 Dec 14;12(12):e0188975. doi: 10.1371/journal.pone.0188975 (PMC5730188; doi:10.1371/journal.pone.0188975)

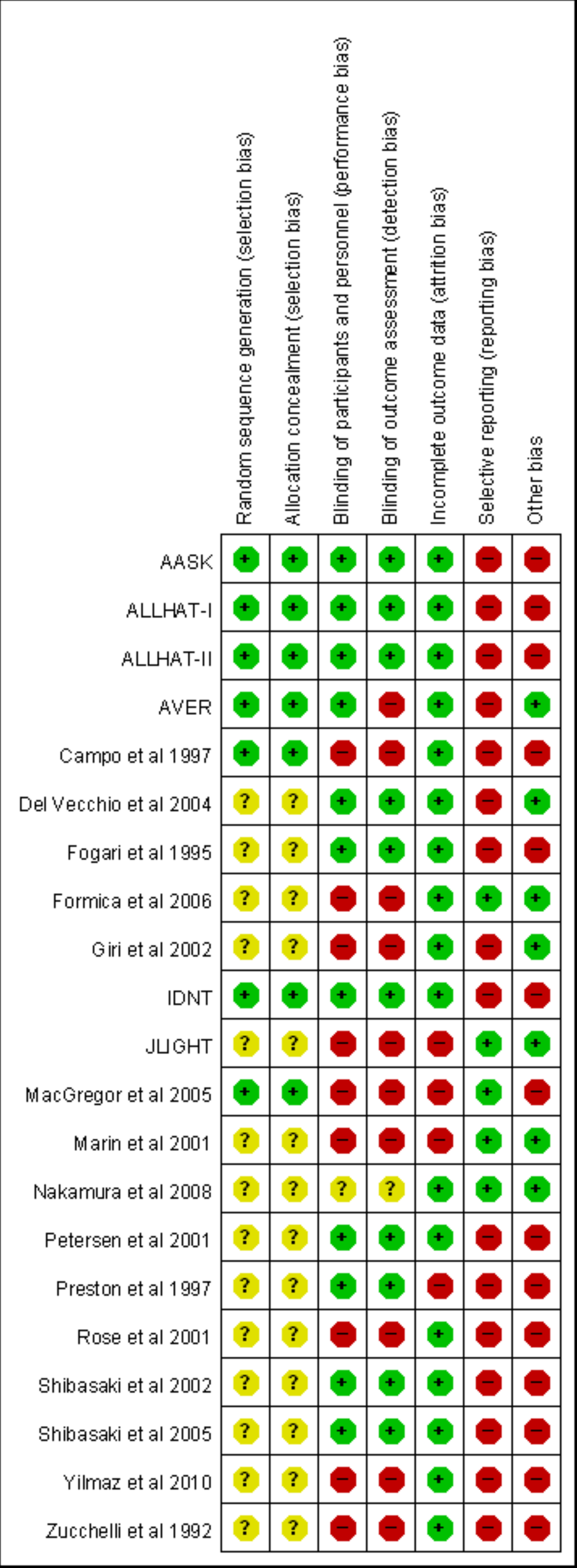

Supplement: S1 Fig — (TIF) [file pone.0188975.s005.tif]

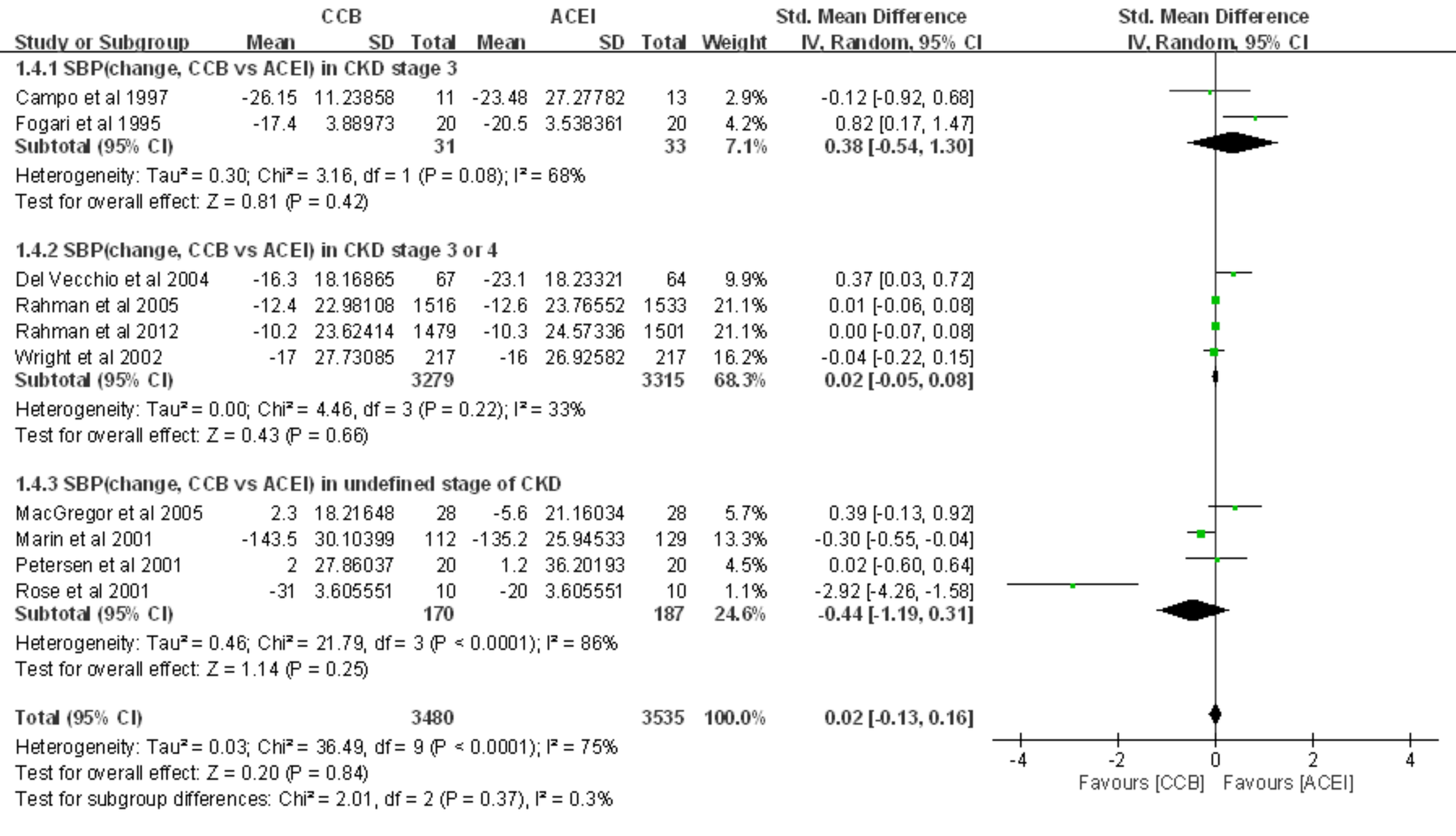

Supplement: S2 Fig — (TIF) [file pone.0188975.s006.tif]

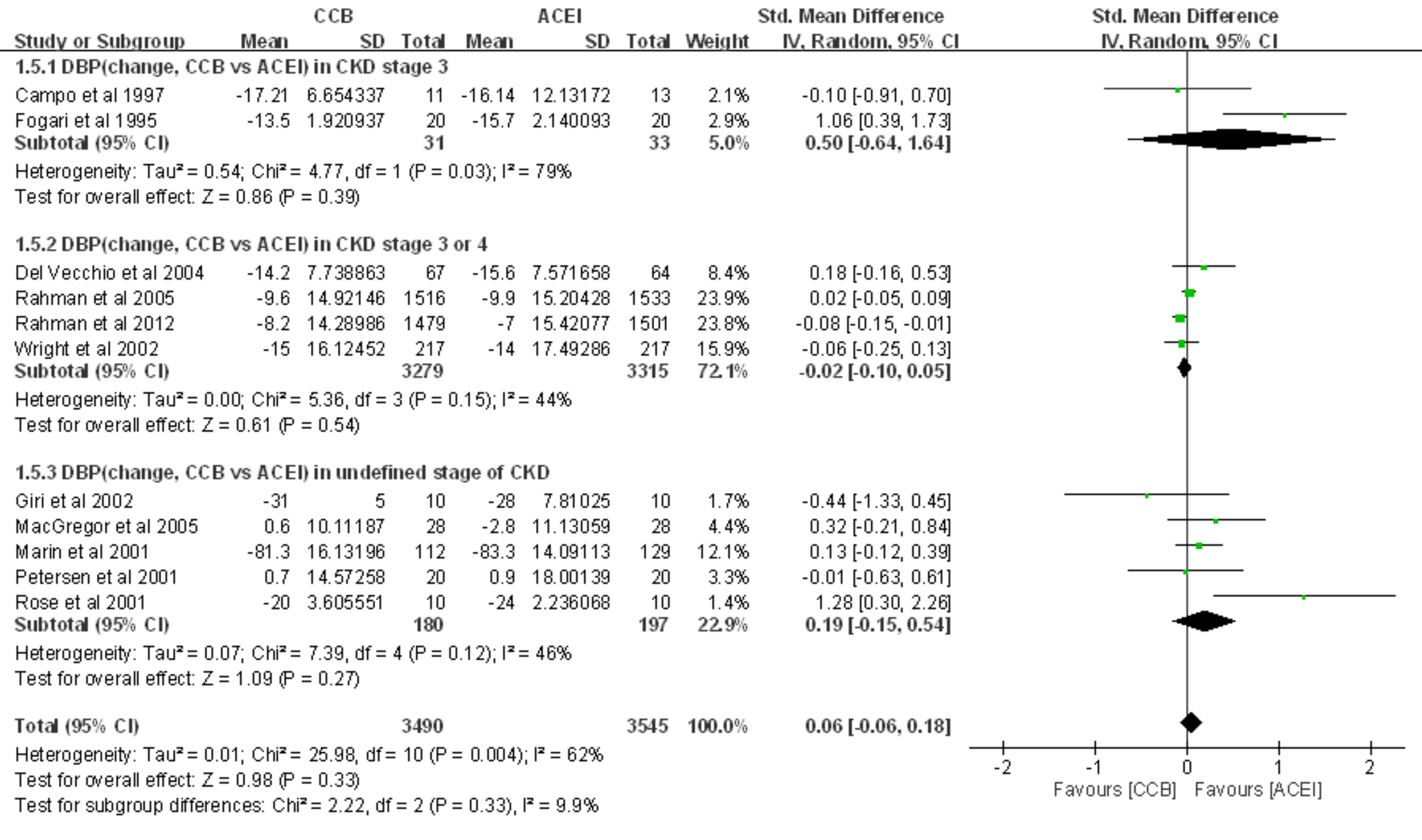

Supplement: S3 Fig — (TIF) [file pone.0188975.s007.tif]

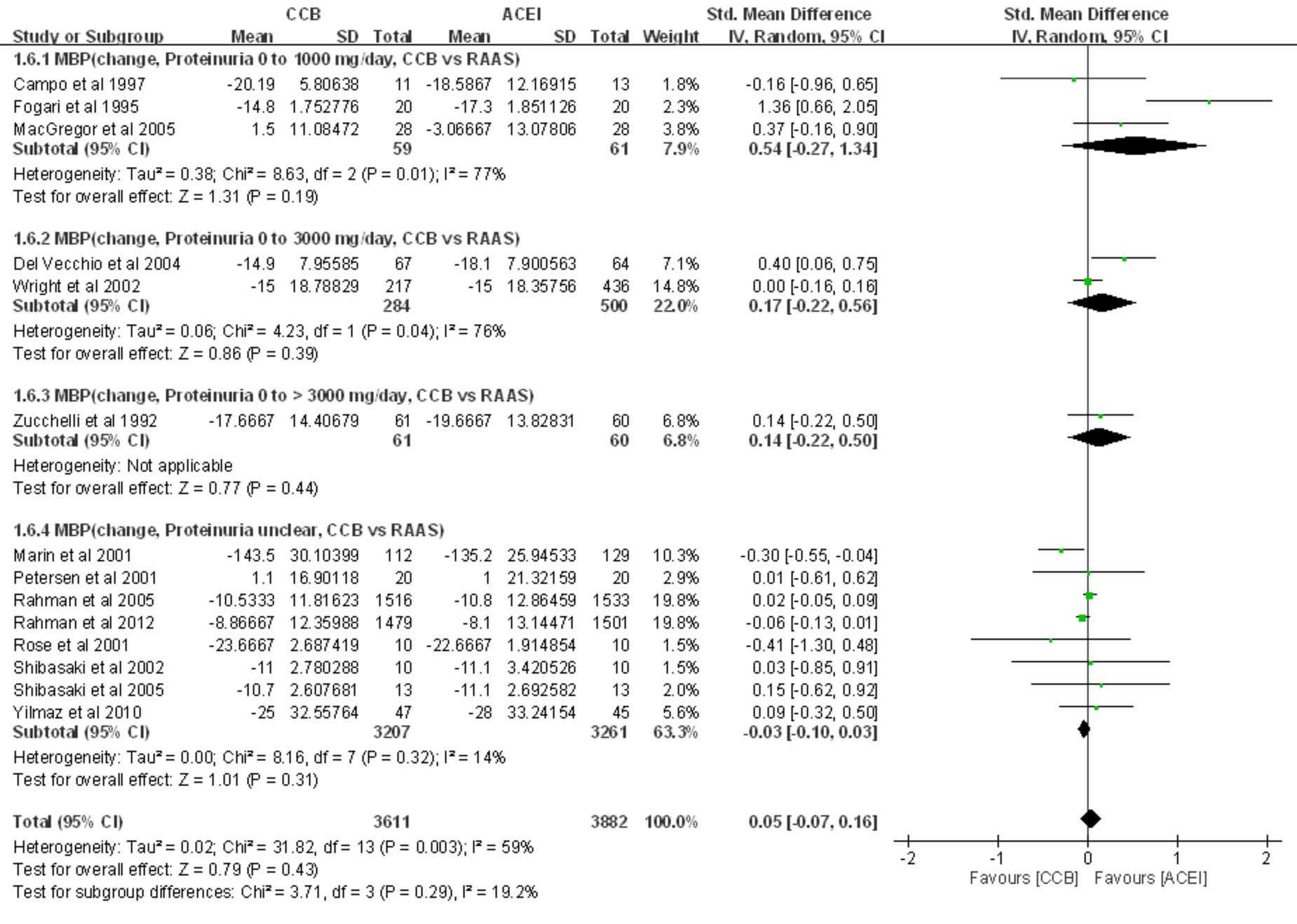

Supplement: S4 Fig — (TIF) [file pone.0188975.s008.tif]

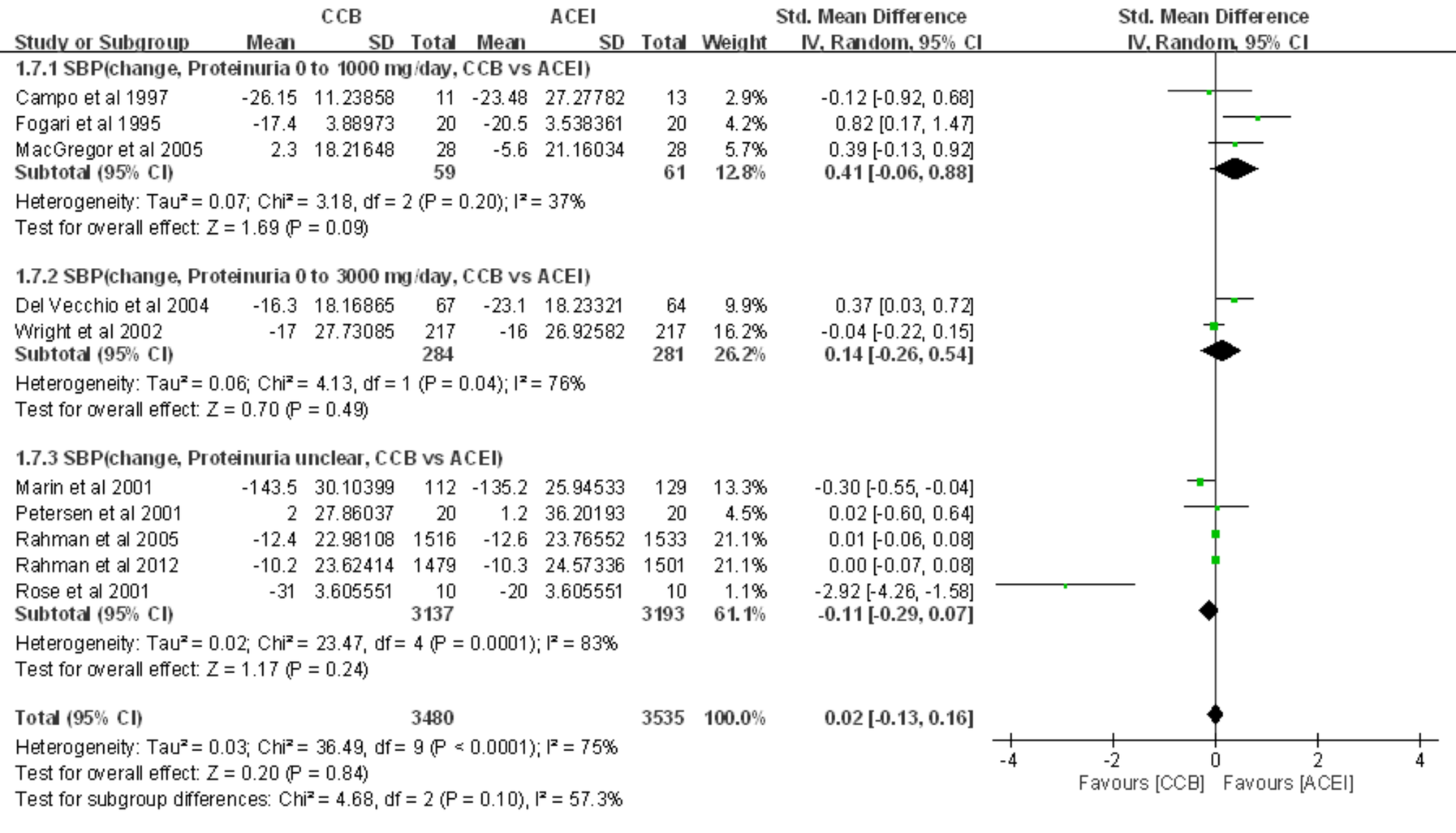

Supplement: S5 Fig — (TIF) [file pone.0188975.s009.tif]

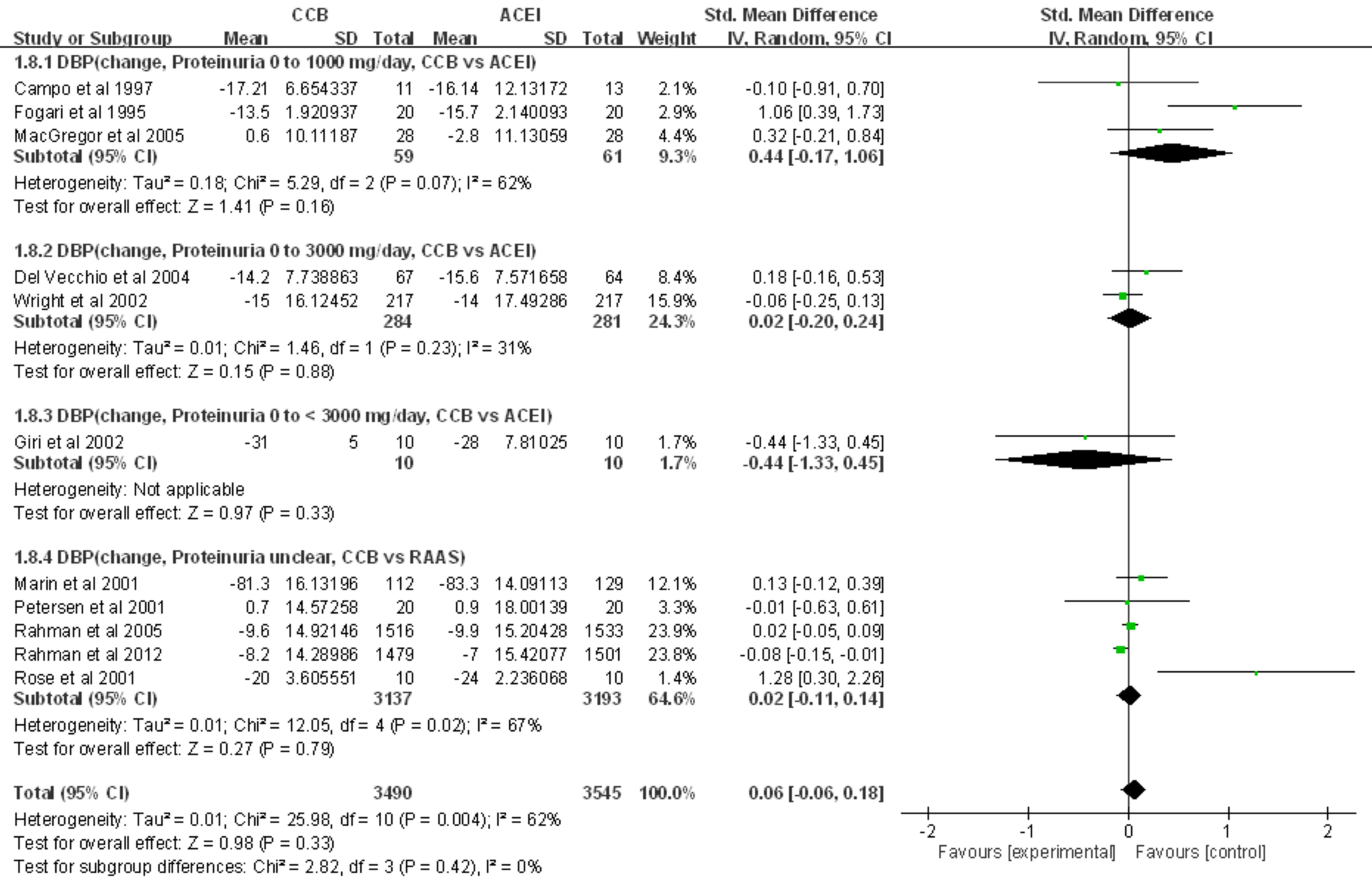

Supplement: S6 Fig — (TIF) [file pone.0188975.s010.tif]

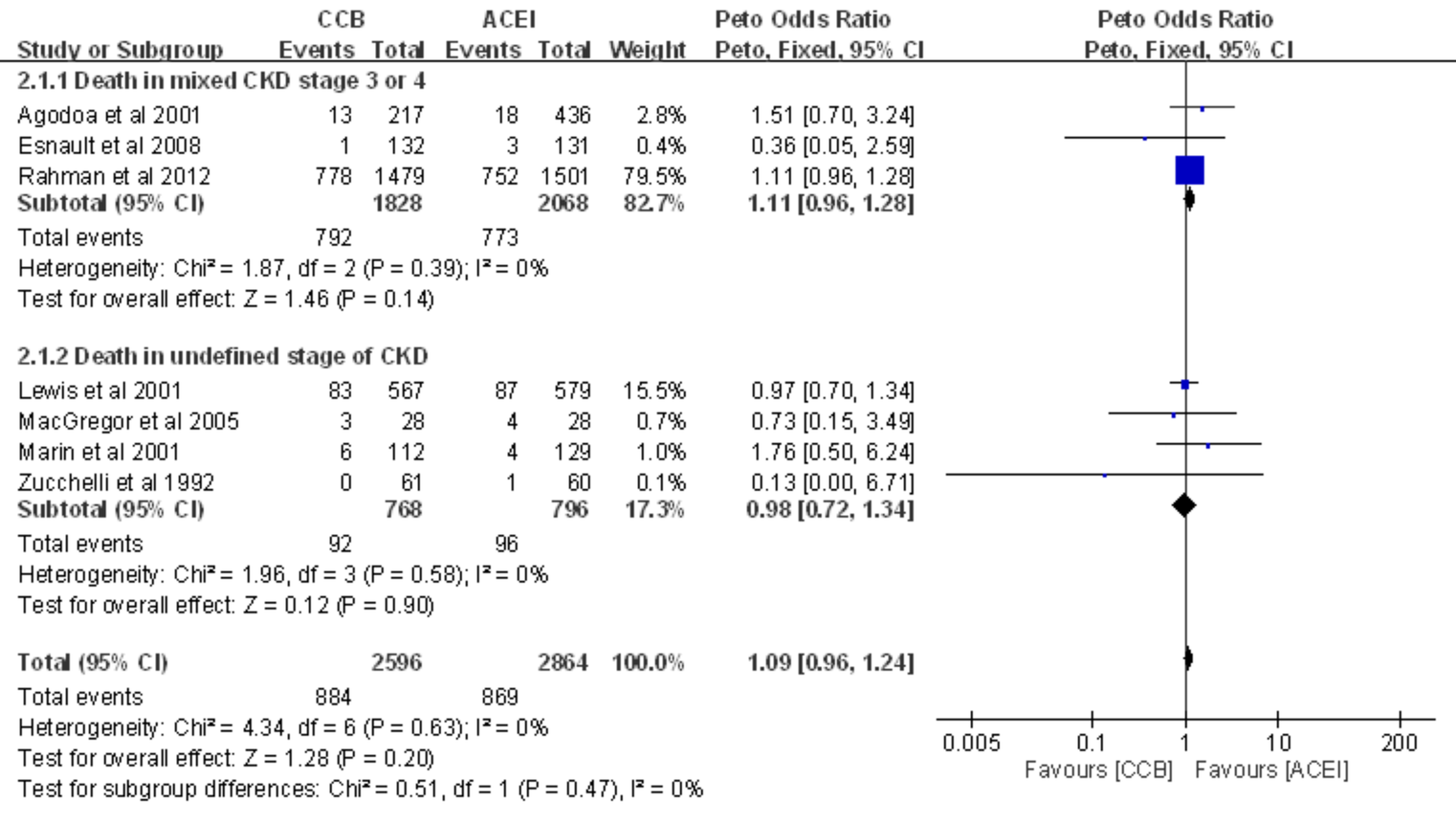

Supplement: S7 Fig — (TIF) [file pone.0188975.s011.tif]

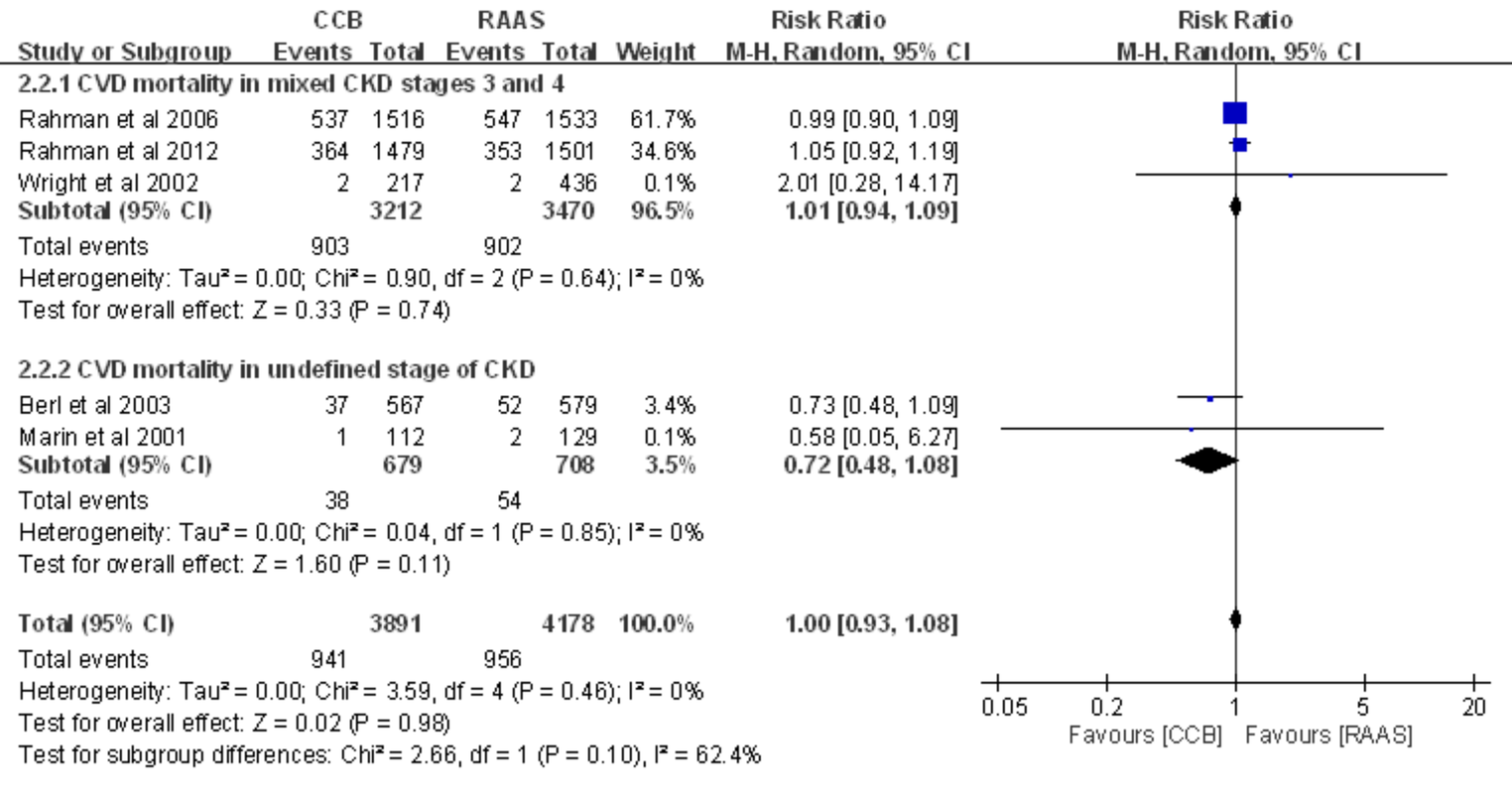

Supplement: S8 Fig — (TIF) [file pone.0188975.s012.tif]

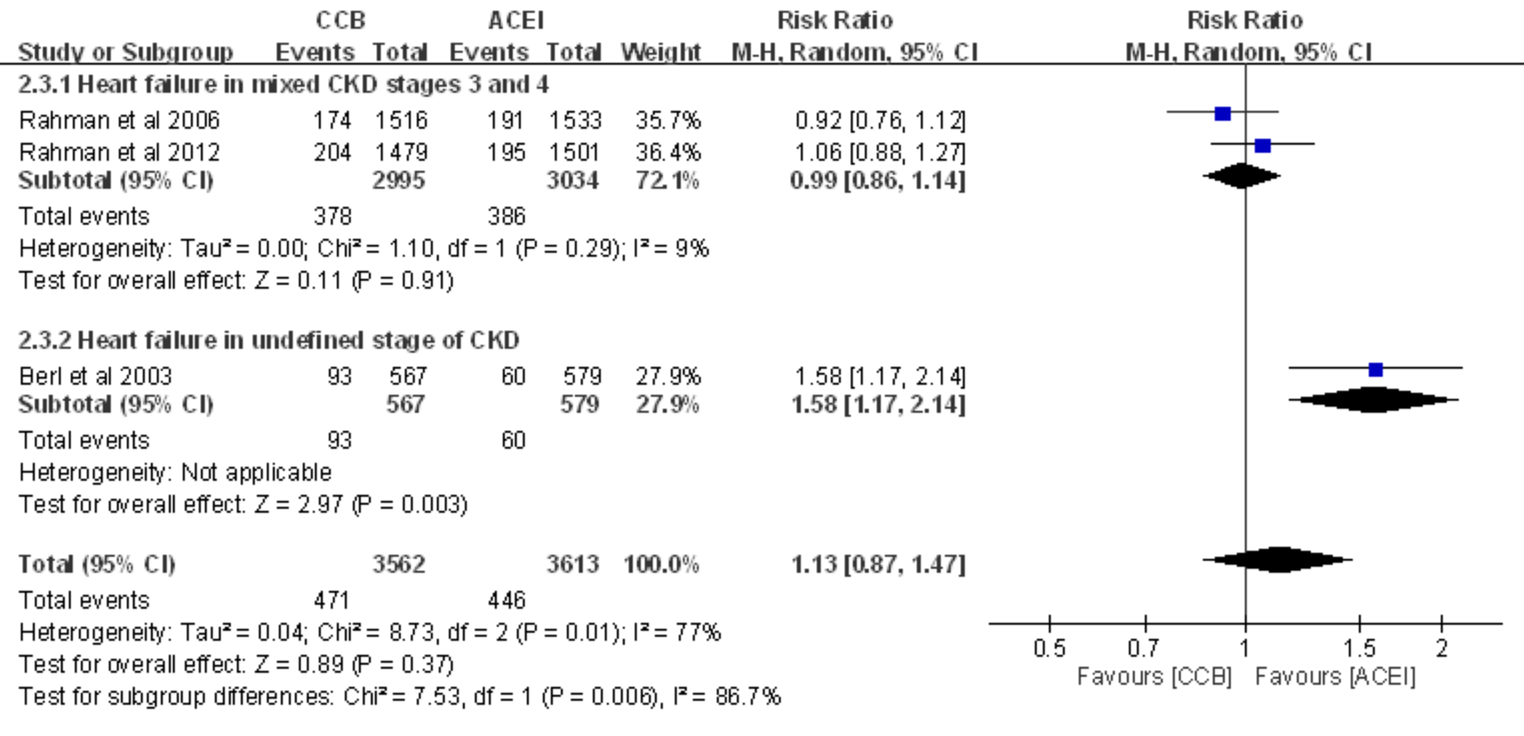

Supplement: S9 Fig — (TIF) [file pone.0188975.s013.tif]

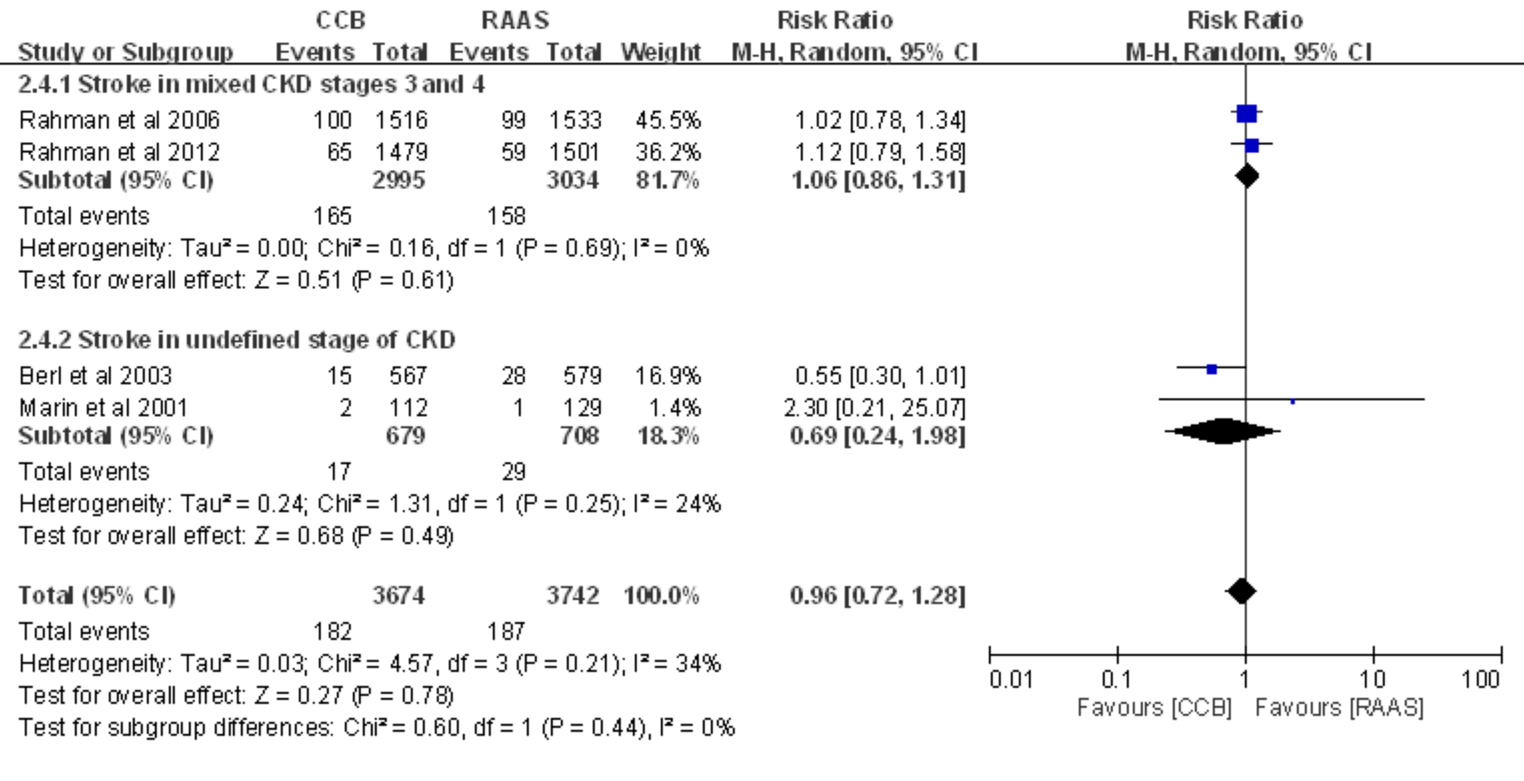

Supplement: S10 Fig — (TIF) [file pone.0188975.s014.tif]

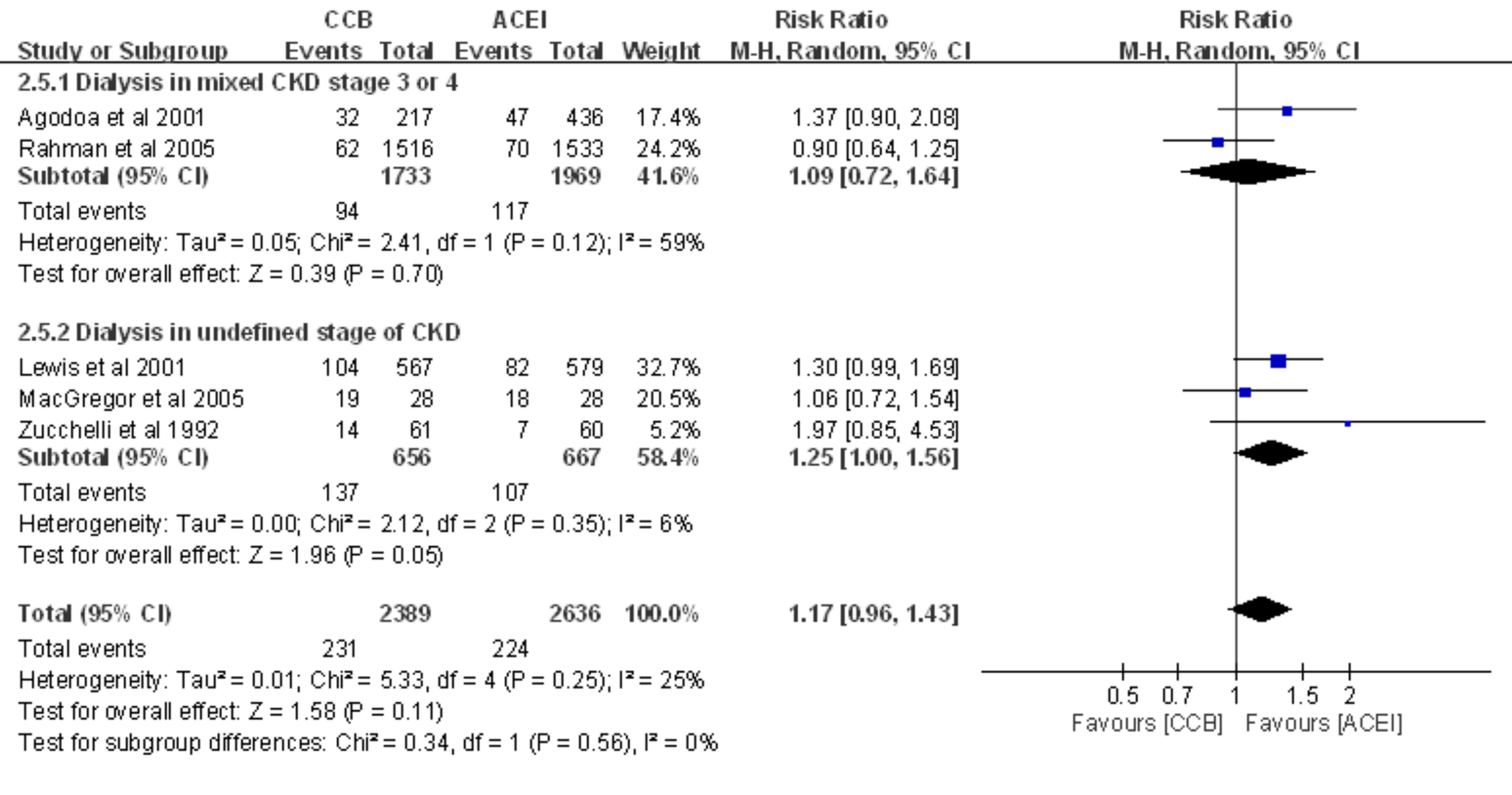

Supplement: S11 Fig — (TIF) [file pone.0188975.s015.tif]

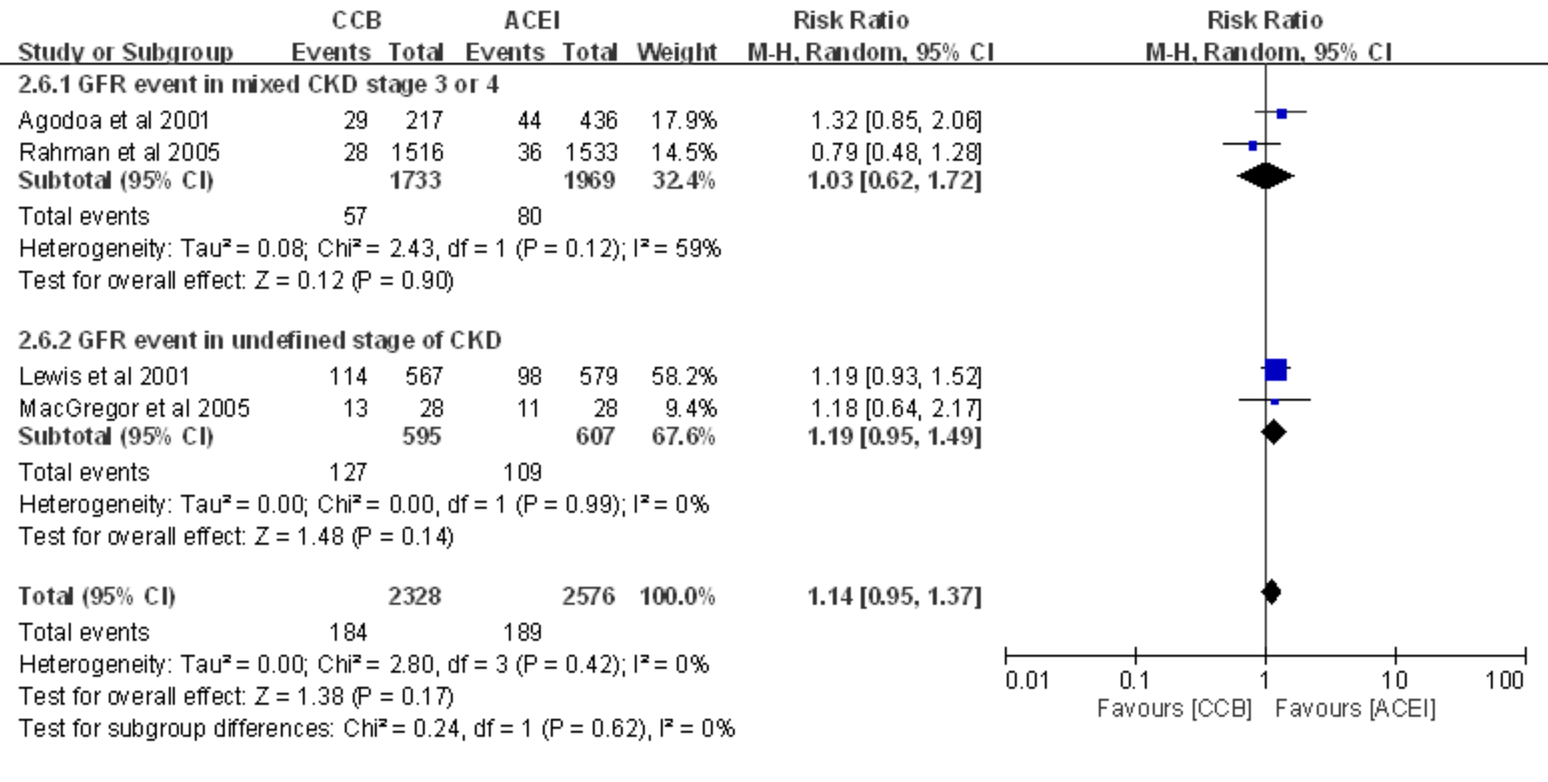

Supplement: S12 Fig — (TIF) [file pone.0188975.s016.tif]

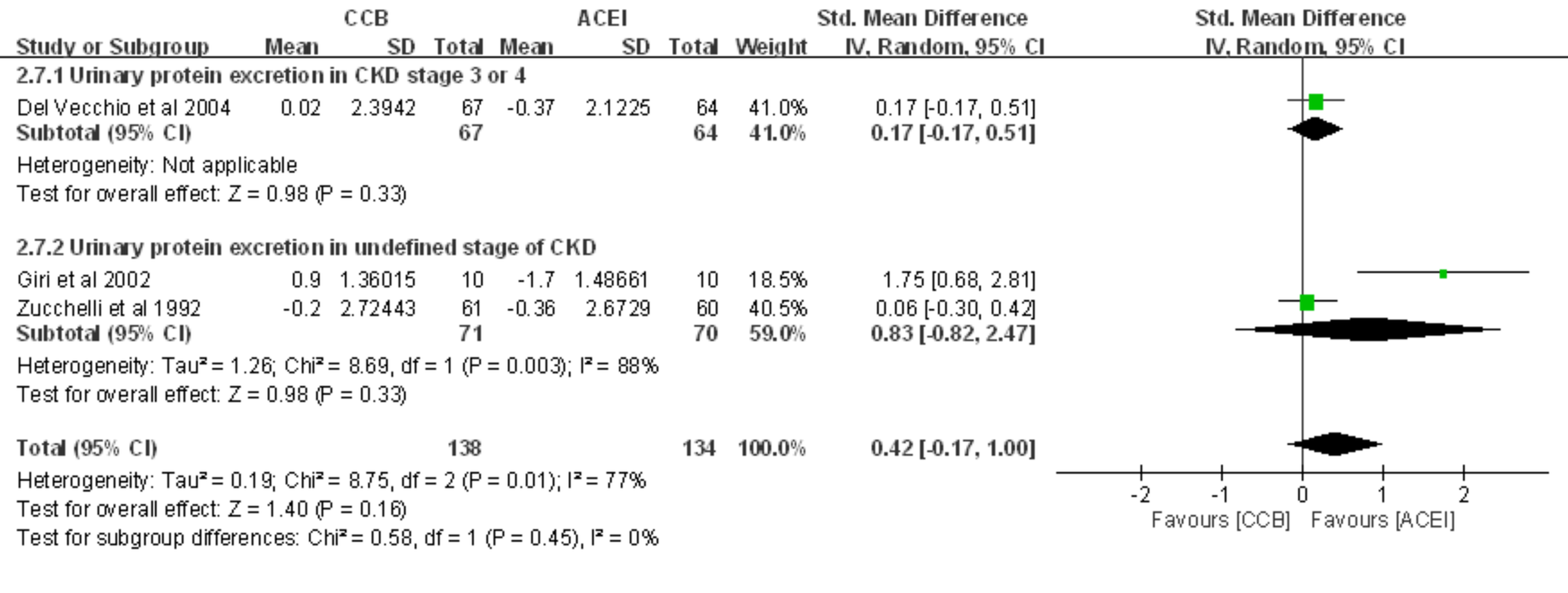

Supplement: S13 Fig — (TIF) [file pone.0188975.s017.tif]
